# CCT2 Promotes Prostate Cancer Progression Through EIF3F‐Dependent Stabilization of FASN

**DOI:** 10.1002/advs.75915

**Published:** 2026-06-03

**Authors:** Shun Xu, Yifan Zhang, Haolin Li, Shengyu Zhao, Xiaoran Dai, Qili Xu, Mintian Fei, Chun Li, Zhihui Zou, Baojun Wang, Li Zhang, Hui Wang, Ligang Zhang, Chaozhao Liang

**Affiliations:** ^1^ Department of Urology, the First Affiliated Hospital of Anhui Medical University Anhui Medical University Hefei Anhui China; ^2^ Institute of Urology Anhui Medical University Hefei Anhui China; ^3^ Anhui Province Key Laboratory of Urological and Andrological Diseases Research and Medical Transformation Anhui Medical University Hefei Anhui China; ^4^ Department of Clinical Laboratory The First Affiliated Hospital of Anhui Medical University Hefei China; ^5^ Department of Urology The First Affiliated Hospital of Kunming Medical University Kunming Yunnan China

**Keywords:** CCT2, EIF3F, FASN, lipid metabolism reprogramming, prostate cancer

## Abstract

Prostate cancer (PCa) is increasingly recognized to be driven by dysregulated lipid metabolism. Although fatty acid synthase (FASN) is highly expressed in PCa, the mechanisms governing FASN protein stability and its functional integration into oncogenic lipid metabolism remain poorly defined. In this study, we identified chaperonin‐containing TCP1 subunit 2 (CCT2) as a key oncogenic regulator that promotes lipid synthesis and enhances malignant phenotypes both in vitro and in vivo. Mechanistically, CCT2 transcription is upregulated by the transcription factor Forkhead Box A1 (FOXA1); the CCT2 protein interacts with eukaryotic translation initiation factor 3 subunit F (EIF3F) and FASN to facilitate the assembly of a CCT2/EIF3F/FASN ternary complex. This complex enhances the EIF3F‐mediated deubiquitination of FASN, increasing FASN stability and lipid synthesis, and accelerating tumor progression. Either orlistat‐mediated FASN inhibition or Y043‐8015‐induced disruption of the CCT2‐EIF3F interaction effectively suppressed CCT2‐driven tumor progression in vivo. Importantly, combined treatment produced synergistic antitumor effects, significantly reducing tumor growth and metastatic burden across multiple in vivo models, including isograft and patient‐derived xenograft models. This study reveals that CCT2 promotes lipid metabolic reprogramming and tumor progression in prostate cancer by cooperating with EIF3F to stabilize FASN, highlighting the CCT2‐EIF3F‐FASN axis as a potential target for metabolic intervention.

## Abstract 2

FOXA1 functions as a transcriptional activator of CCT2, increasing its mRNA expression in PCa cells. Upregulated CCT2 interacts with EIF3F, forming a ternary complex with FASN. This complex enhances the EIF3F‐mediated deubiquitination of FASN, preventing its proteasomal degradation. Stabilized FASN promotes fatty acid synthesis and lipid droplet accumulation, which are associated with increased tumor cell proliferation and metastatic phenotypes observed in vivo.

## 1 Introduction

Prostate cancer (PCa) ranks as the fourth most common malignancy worldwide and the fifth leading cause of cancer‐related death in men [1]. Although the oncologic outcomes of localized PCa following definitive treatment are favourable (5‐year survival exceeding 99% with surgical resection or radiotherapy) [2], advanced disease is frequently associated with disease progression and metastatic dissemination, posing substantial therapeutic challenges [3, 4]. Androgen deprivation therapy (ADT) remains the cornerstone of treatment for advanced and metastatic PCa. Although ADT is initially effective in controlling tumor progression, most patients ultimately develop resistance to ADT, leading to disease relapse and poor clinical outcomes [5]. Consequently, there is an urgent need to elucidate the molecular mechanisms underlying PCa progression to provide a theoretical framework and practical basis for the progression of novel therapeutic strategies.

Molecular chaperones are essential for maintaining proteostasis, facilitating protein folding, protein complex assembly, and targeted protein degradation. Conversely, chaperone protein dysfunction can lead to the accumulation of misfolded or aggregated proteins [6]. Chaperonin‐containing TCP1 subunit 2 (CCT2), a core component of the TRiC/CCT complex, mediates the proper folding of cytosolic proteins in an ATP‐dependent manner [7]. Beyond its canonical chaperone function, CCT2 has also been implicated in the regulation of the cell cycle [8]. Interestingly, CCT2 is overexpressed in various malignancies, including colorectal, ovarian, and breast cancers, and has been shown to play a critical role in the regulation of protein ubiquitination. By modulating the stability of multiple oncogenic proteins [9, 10, 11], CCT2 extends the functional scope of molecular chaperones beyond protein folding, positioning it as a potential regulatory factor in tumor progression. However, whether CCT2 plays a similar regulatory role in PCa and how it influences tumor progression remain unclear, highlighting the need for further mechanistic investigation.

Aberrant lipid metabolism is a hallmark of PCa. Fatty acid synthase (FASN), the rate‐limiting enzyme in de novo fatty acid synthesis, is transcriptionally regulated by multiple transcription factors, including SREBP1 [12, 13, 14], c‐Myc [15, 16], FOXM1 [17], LXR [18], ChREBP [19], and the androgen receptor (AR) [20]. FASN‐mediated fatty acid synthesis has been widely implicated in PCa progression [21, 22, 23], as supported by RNA sequencing and targeted lipidomic analyses. Although the oncogenic role of FASN is well established, the mechanisms governing its protein stability in PCa remain largely unexplored. Our mass spectrometry analysis revealed an interaction between CCT2 and FASN, suggesting that CCT2 may participate in the post‐translational regulation of lipid metabolism through non‐canonical mechanisms.

Eukaryotic translation initiation factor 3 (EIF3) is a multi‐subunit complex that is essential for the initiation of protein translation in eukaryotic cells. Specifically, EIF3 orchestrates the assembly of the translation initiation machinery [24, 25]. As a component of the EIF3 complex, EIF3F primarily contributes to maintaining the structural integrity of the complex. However, emerging evidence indicates that EIF3F can dissociate from EIF3 under specific conditions and exert independent biological functions. For example, in pancreatic cancer and melanoma cells, EIF3F has been shown to promote apoptosis independently of its association with the EIF3 complex [26]. In colorectal cancer, EIF3F mediates deubiquitination through its MPN domain, thereby influencing reprogramming of the serine–glycine–one‐carbon (SGOC) metabolic pathway [27]. Notably, EIF3F is also expressed at high levels in PCa [28]. Mass spectrometry analysis further revealed an interaction between EIF3F and FASN, suggesting that EIF3F may participate in the post‐translational regulation of FASN through a similar deubiquitination mechanism. However, this possibility warrants further validation.

In this study, we investigated how CCT2 contributes to PCa progression by reprogramming lipid metabolism. Forkhead Box A1 (FOXA1), a key oncogenic transcription factor, was identified for the first time as a direct transcriptional activator of CCT2. Although FASN‐driven lipid metabolism has been widely implicated in tumor progression, the regulatory mechanisms governing FASN protein stability remain poorly understood. Our findings demonstrated that CCT2 forms a complex with EIF3F and FASN, enhancing FASN stability by inhibiting FASN ubiquitination. Subsequent studies revealed that Y043‐8015, a small molecule that can specifically disrupt the interaction between CCT2 and EIF3F, suppressed PCa cell growth. Tumor growth and metastatic phenotypes associated with CCT2 activity were substantially attenuated by orlistat administration. Notably, combined treatment with Y043‐8015 and orlistat exerted enhanced anti‐tumor effects compared with either agent alone. On the basis of these findings, we uncover a previously unrecognized regulatory mechanism in which CCT2 functions as a molecular scaffold, linking the deubiquitination activity of EIF3F to the stabilization of FASN. This study not only provides mechanistic insight into the FOXA1‐CCT2‐EIF3F‐FASN signaling axis in the metabolic reprogramming of PCa but also offers a potential strategy for targeted therapeutic intervention.

## 2 Results

### 2.1 CCT2 Expression is Significantly Upregulated in PCa and is Positively Correlated With Tumor Progression and Poor Clinical Prognosis

**FIGURE 1:**
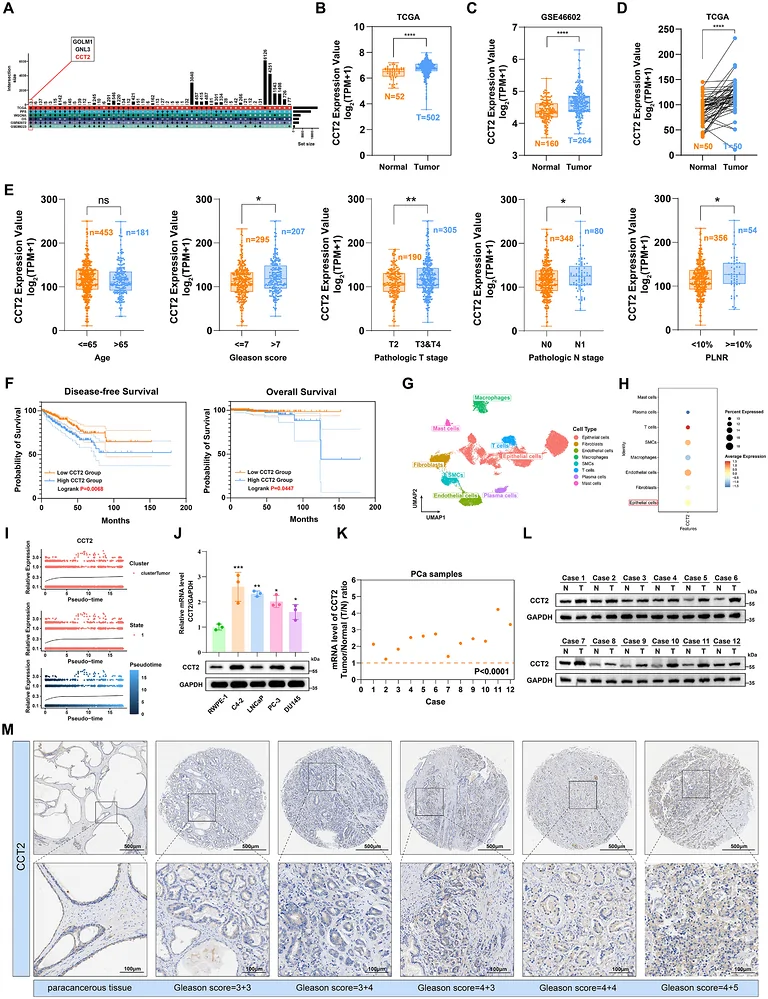
CCT2 is upregulated in prostate cancer and is correlated with poor prognosis. (A) Integrative analysis of six datasets identified three upregulated genes associated with prostate cancer (PCa) progression and prognosis (CCT2, GNL3, and GOLM1). (B,C) Comparison of CCT2 expression in tumor and normal tissues in the TCGA and GSE46602 datasets. Sample sizes (n) for each group are indicated in the plots. Statistical significance was assessed using two‐tailed unpaired Student's *t*‐tests. (D) Paired comparison of CCT2 expression in matched tumor and normal tissues from the TCGA dataset. Sample size (n) is indicated in the plot. Statistical significance was assessed using a paired two‐tailed Student's *t*‐test. (E) Box‐and‐whisker plots with individual data points showing the association of CCT2 expression with age, Gleason score, pathologic T stage, pathologic N stage, and pelvic lymph node ratio (PLNR). Sample sizes (n) are indicated in each plot. Statistical significance was assessed using two‐tailed unpaired Student's *t*‐tests for each comparison. (F) Kaplan‐Meier survival curves were generated to evaluate the association between CCT2 expression levels and DFS and OS in patients with PCa from TCGA dataset. Statistical significance was determined using the log‐rank test. (G) UMAP plot from single‐cell RNA sequencing (GSE206962) reveals eight distinct cell clusters in the TME. (H)Dot plot showing the percentage of CCT2‐expressing cells and average CCT2 expression levels across different cell types. (I) Pseudotime trajectory analysis revealed a progressive increase in CCT2 expression during cellular differentiation. (J) qRT–PCR and Western blotting analyses of CCT2 expression in normal prostate epithelial cells (RWPE‐1) and prostate cancer cell lines (C4‐2, LNCaP, PC‐3, and DU145). Quantitative data are presented as mean ± SD from three independent biological replicates (n = 3). Statistical significance was assessed using one‐way ANOVA. (K) qRT‐PCR analysis of CCT2 mRNA expression in paired prostate cancer (T) and adjacent normal (N) tissue samples (n = 12 pairs). Data are presented as tumor‐to‐normal (T/N) ratios. Statistical significance was determined using a paired two‐tailed Student's *t*‐test. (L) Representative Western blotting images showing CCT2 protein expression in paired tumor (T) and adjacent normal (N) prostate tissues (n = 12 pairs). GAPDH was used as a loading control. (M) Representative immunohistochemistry images of CCT2 in paracancerous tissues and PCa tissues with various Gleason scores. Scale bars: upper panel, 500 µm; lower panel, 100 µm. For quantitative panels with significance annotations, ns indicates *p* > 0.05, *p < 0.05, **p < 0.01, ***p < 0.001, ****p < 0.0001.

To determine the clinical significance of CCT2 in PCa, we performed integrated bioinformatic analyses and validated its expression and prognostic relevance using patient samples and TMA. WGCNA was performed to identify tumor‐related gene modules in TCGA (Figure S1A, Supporting Information). By intersecting the WGCNA results with datasets from TCGA, progression‐free survival (PFS) data, overall survival (OS) data, and the GEO datasets GSE62872 and GSE89223, three candidate genes were identified: GOLM1, GNL3, and CCT2 (Figure 1A). Among these genes, the associations of GOLM1 [29, 30] and GNL3 [31] with PCa have been previously reported. Therefore, CCT2 was selected for further investigation.

To assess CCT2 expression in PCa, we compared the levels of CCT2 expression in PCa tissues and normal tissues in the TCGA database and the GSE46602 dataset. Our results demonstrated that CCT2 was significantly overexpressed in PCa tissues (Figure 1B–D). Further analysis of the TCGA database revealed that elevated CCT2 expression was significantly associated with adverse clinicopathological parameters, including a higher Gleason score, advanced pathological T stage, a positive pathological N stage, and an increased pelvic lymph node ratio (PLNR), but not with patient age (Figure 1E). Using the median CCT2 expression level from TCGA data as a cut‐off, we divided patients into a high‐expression group and a low‐expression group. Kaplan‐Meier analysis revealed significantly shorter disease‐free survival (DFS) and overall survival (OS) in the high CCT2 expression group (Figure 1F). These findings strongly suggest that CCT2 overexpression is closely linked to tumor aggressiveness and poor prognosis in PCa patients.

To comprehensively investigate the role of CCT2 in PCa pathogenesis, we reanalyzed the GSE206962 PCa single‐cell RNA sequencing dataset. We identified and annotated eight distinct cell populations (Figure 1G and Figure S1B, Supporting Information). Single‐cell expression analysis revealed high CCT2 expression in epithelial cells, endothelial cells, and T cells, as demonstrated by both the percentage of cells expressing CCT2 and the average CCT2 expression level (Figure 1H). To delineate the dynamic expression pattern of CCT2 during cellular differentiation, we performed pseudotime trajectory analysis of the scRNA‐seq data. The results demonstrated the progressive upregulation of CCT2 expression along the differentiation trajectory, reaching a plateau at terminal differentiation stages (Figure 1I). This expression pattern suggests a potential oncogenic role of CCT2 in tumor progression.

To validate the above bioinformatics findings, we quantified CCT2 expression in multiple PCa cell lines (C4‐2, LNCaP, PC‐3, and DU145) and clinical specimens using qRT‐PCR and Western blotting analysis. Compared with those in normal prostate epithelial cells (RWPE‐1) and matched paracancerous tissues, CCT2 expression was markedly upregulated at both the mRNA and protein levels in PCa models (Figure 1J–L and Figure S1C,D, Supporting Information), supporting the potential of CCT2 as a PCa biomarker. Similarly, immunohistochemical analysis of TMAs revealed significantly elevated CCT2 expression in PCa tissues compared with normal prostate tissues. Notably, CCT2 expression levels progressively increased with increasing Gleason score (Figure 1M). Chi‐square tests revealed significant positive correlations between high CCT2 expression and adverse clinicopathological parameters, including an advanced Gleason score (*p* < 0.001), a higher Gleason grade group (P = 0.045), an advanced T stage (P = 0.003), and poorer survival status (P = 0.018) (Table S1, Supporting Information). Collectively, these findings demonstrate that CCT2 represents a promising independent prognostic biomarker for clinical outcomes and may play a critical role in PCa progression.

### 2.2 CCT2 Mediates Adipogenic Reprogramming to Promote PCa Progression In Vitro

**FIGURE 2:**
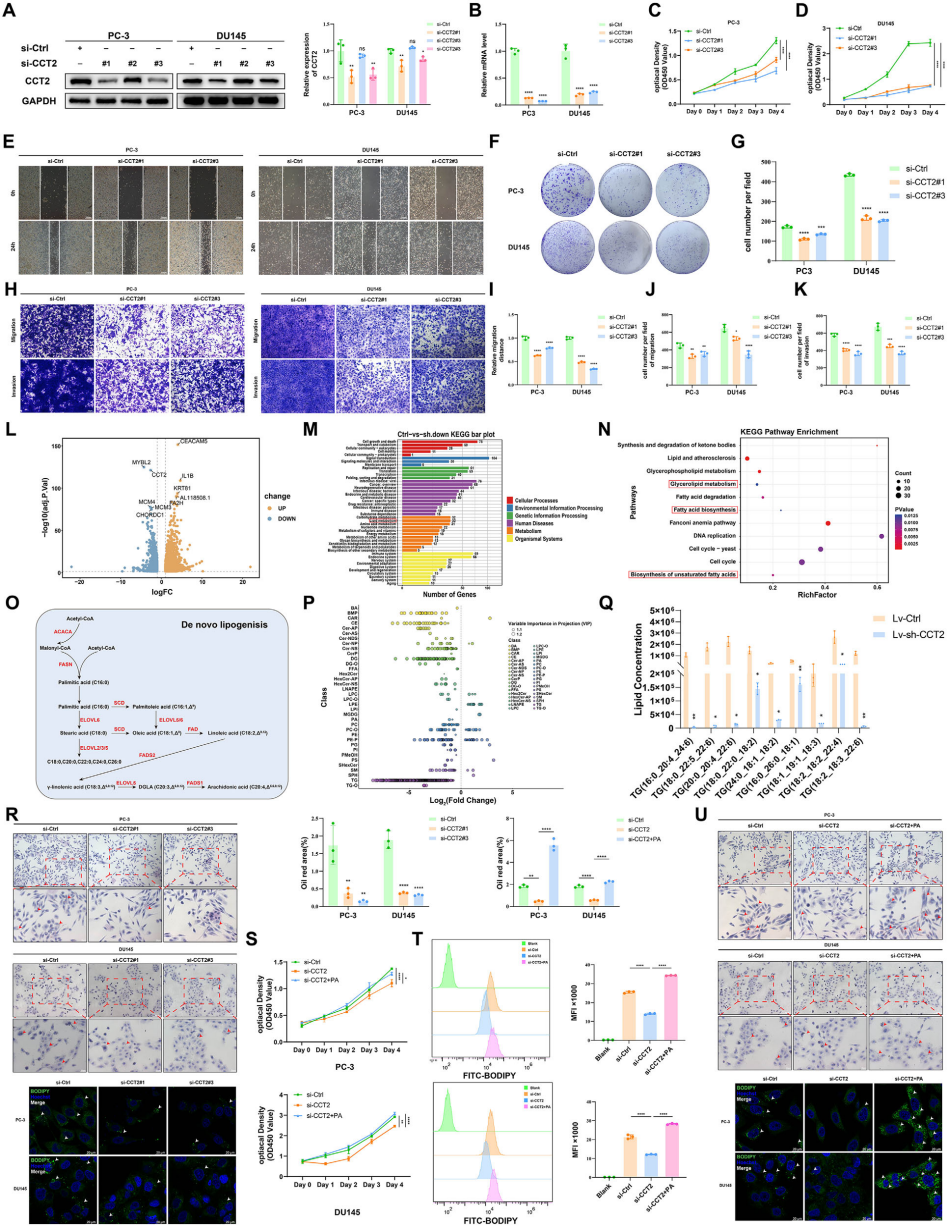
CCT2 mediates adipogenic reprogramming to promote PCa progression in vitro. (A,B) qRT‒PCR and Western blotting analyses confirming the efficiency of CCT2 knockdown in PC‐3 and DU145 cells. Quantitative data are presented as mean ± SD from three independent biological replicates (n = 3). Statistical significance was assessed using one‐way ANOVA. (C,D,F,G) CCK‐8 and colony formation assays were performed to evaluate the effects of CCT2 knockdown on the viability and proliferative capacity of PC‐3 and DU145 cells. Data are presented as mean ± SD from three independent biological replicates (n = 3). Statistical analysis was performed using two‐way ANOVA for CCK‐8 growth curves and one‐way ANOVA for colony formation quantification. (E,H–K) Wound healing and Transwell assays were conducted to assess the effects of CCT2 knockdown on the migratory and invasive behavior of PC‐3 and DU145 cells. Data are presented as mean ± SD from three independent biological replicates (n = 3). Statistical significance was assessed using one‐way ANOVA. (L) Volcano plot of differentially expressed genes (DEGs) between Ctrl and sh‐CCT2 PC‐3 cells based on RNA‐seq. (M,N) KEGG pathway enrichment analyses revealed significant enrichment in lipid biosynthesis and metabolic pathways among the DEGs. (O) Schematic diagram illustrating the de novo lipogenesis pathway. (P,Q) Lipidomic profiling was performed to examine changes in triglyceride (TG) levels following CCT2 knockdown in PC‐3 cells. Data are presented as mean ± SD from three independent biological replicates (n = 3). Statistical significance was assessed using two‐tailed unpaired Student's *t*‐tests. (R) Oil Red O and BODIPY 493/503 staining were used to visualize intracellular lipid droplets in control and CCT2‐knockdown. Data are presented as mean ± SD from three independent biological replicates (n = 3). Statistical significance for the quantification of Oil Red O staining was assessed using one‐way ANOVA. (S‐U) Lipid staining and CCK‐8 assays were conducted to evaluate the effects of exogenous PA supplementation on lipid content and cell viability in CCT2‐deficient PCa cells. Data are presented as mean ± SD from three independent biological replicates (n = 3). Statistical significance was assessed using two‐way ANOVA for growth curves, and one‐way ANOVA for quantitative analyses of lipid droplet content, including flow cytometry–based lipid quantification and Oil Red O staining. For quantitative panels with significance annotations, ns indicates *p* > 0.05, *p < 0.05, **p < 0.01, ***p < 0.001, ****p < 0.0001.

Having identified a correlation between CCT2 and PCa progression, we next sought to investigate its functional role in PCa. We genetically knocked down and overexpressed CCT2 using siRNA and a pcDNA3.1 overexpression construct, respectively, in PCa cells; the transfection efficiency was verified through qRT‐PCR and Western blotting analysis (Figure 2A,B and Figure S2A,B, Supporting Information). The role of CCT2 in regulating the proliferative capacity of PCa cells was assessed using CCK‐8 (to evaluate short‐term proliferation) and colony formation (to evaluate long‐term clonogenic survival) assays. CCT2 knockdown significantly suppressed cell proliferation (Figure 2C,D) and reduced colony‐forming ability (Figure 2F,G), whereas CCT2 overexpression enhanced cell proliferation and colony‐forming ability (Figure S2C,D,F,G, Supporting Information). Wound healing assays demonstrated that CCT2 knockdown significantly impaired cell motility, as evidenced by a reduced 24‐h wound closure rate compared with that of the control (Figure 2E,I). Consistent with these findings, Transwell migration and Matrigel invasion assays revealed marked decreases in both migratory and invasive cell populations following CCT2 knockdown (Figure 2H,J,K). CCT2 overexpression had the opposite effects (Figure S2E,H,I–K, Supporting Information), indicating increased metastatic potential. Collectively, these results establish CCT2 as a critical regulator of PCa cell proliferation and metastatic behavior, with its expression levels directly correlated with malignant progression.

Given the pronounced phenotypic alterations observed following CCT2 knockdown, we performed RNA‐seq of PC‐3 cells transfected with Lv‐sh‐CCT2 and Lv‐Ctrl to elucidate the underlying molecular mechanisms. The shRNA sequence targeting CCT2 was validated, and efficient knockdown of CCT2 expression was confirmed at the protein level by western blotting (Figure S2L, Supporting Information). Functional assays further demonstrated that lentiviral‐mediated CCT2 knockdown markedly suppressed the proliferation of PC‐3 cells, as assessed by cell growth curves (Figure S2L, Supporting Information). A comparison of the two datasets revealed 4602 differentially expressed genes (DEGs), with 2911 significantly upregulated and 1691 downregulated genes (Figure 2L) upon CCT2 knockdown. GO and KEGG analyses revealed significant enrichment in genes associated with the synthesis of fatty acids in lipid metabolism (Figure 2M,N and Figure S2M, Supporting Information). The de novo fatty acid synthesis pathway is illustrated in a schematic diagram (Figure 2O). These findings suggest that CCT2 may modulate PCa progression through the regulation of lipid metabolic pathways.

To further investigate CCT2‐mediated metabolic reprogramming, we conducted comprehensive targeted lipidomic profiling in PC‐3 cells following CCT2 knockdown. Quantitative analysis revealed 737 lipid species with significantly altered abundance, with 58 metabolites upregulated and 679 downregulated compared to Lv‐Ctrl cells (Figure S2M, Supporting Information). Consistent with the GO enrichment results (Figure S2N, Supporting Information), Lipidomic analysis revealed a significant reduction in multiple triglyceride species following CCT2 knockdown (Figure 2P,Q). The heatmap of differential lipid clustering is shown in Figure S2O, Supporting Information. Oil Red O staining and BODIPY 493/503 fluorescence imaging confirmed that CCT2 knockdown significantly reduced the number of intracellular lipid droplets, whereas CCT2 overexpression increased lipid storage (Figure 2R and Figure S2P,Q, Supporting Information). Interestingly, supplementation with palmitate partially rescued both the lipid depletion phenotype and the proliferation defect in CCT2‐knockdown cells (Figure 2S–U), further establishing the functional link between CCT2‐mediated adipogenesis and PCa progression. In summary, our findings demonstrate that CCT2 serves as a critical regulator of lipid metabolic reprogramming in PCa.

### 2.3 CCT2 Stabilizes FASN Through Physical Interaction and Suppression of Ubiquitin‐Dependent FASN Degradation

**FIGURE 3:**
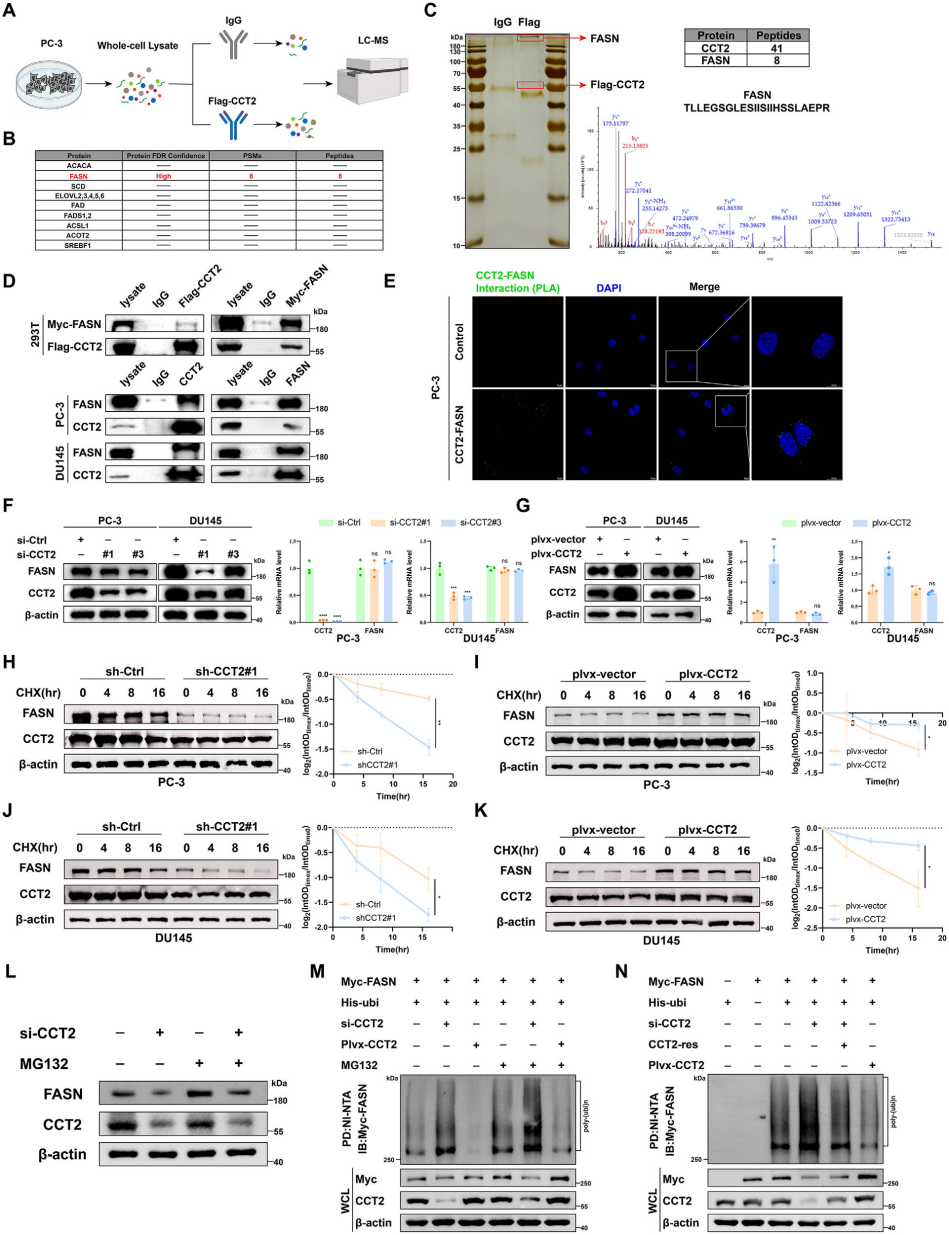
CCT2 stabilizes FASN through physical interaction and suppression of its ubiquitin‐dependent degradation. (A) Schematic workflow of Co‐IP followed by LC‒MS/MS to identify CCT2‐binding proteins. (B) Mass spectrometry analysis was performed to identify proteins interacting with CCT2, with FASN detected as a high‐confidence candidate. (C) Representative MS spectrum showing a unique peptide derived from FASN. (D) Reciprocal Co‐IP assays in HEK‐293T cells and PCa cell lines (PC‐3, DU145) were conducted to validate both exogenous and endogenous interactions between CCT2 and FASN. (E)Proximity ligation assay (PLA) showing in situ interaction between endogenous CCT2 and FASN in PC‐3 cells; nuclei were counterstained with DAPI. (F,G) qRT‐PCR and Western blotting analyses were performed to examine the effects of CCT2 knockdown or overexpression on FASN mRNA and protein levels. Data are presented as mean ± SD from three independent biological replicates (n = 3). Statistical analysis was performed using one‐way ANOVA for CCT2 knockdown and two‐tailed unpaired Student's *t*‐tests for CCT2 overexpression. (H–K) CHX chase assays were used to evaluate FASN protein stability following CCT2 knockdown or overexpression. Representative immunoblots and corresponding protein decay curves are shown. Protein band intensities were quantified by densitometry, normalized to β‐actin, and expressed relative to the 0 h time point. Decay constants (k) were obtained by linear regression of log_2_‐transformed normalized intensities. Data are presented as mean ± SD from three independent biological replicates (n = 3). Statistical significance was determined using a two‐tailed Student's *t*‐test. (L) Western blotting analysis evaluating the effect of the proteasome inhibitor MG132 on FASN protein levels in the presence or absence of CCT2. (M,N) Ni–NTA pull‐down assays detecting ubiquitinated FASN in cells transfected with His‐tagged ubiquitin, demonstrating that CCT2 suppresses FASN ubiquitination. For quantitative panels with significance annotations, ns indicates *p* > 0.05, *p < 0.05, **p < 0.01, ***p < 0.001, ****p < 0.0001.

To elucidate the molecular mechanism underlying CCT2‐regulated lipid synthesis, we performed Co‐IP of Flag‐tagged CCT2 coupled with mass spectrometry (IP‐MS) in PC‐3 cells, which identified FASN as the only lipid metabolism enzyme that interacts with CCT2 (Figure 3A–C). To confirm the physical association between CCT2 and FASN, reciprocal Co‐IP experiments were carried out in exogenous (HEK‐293T) and endogenous (PC‐3, DU145) systems, and the results confirmed the CCT2‐FASN interaction (Figure 3D). Furthermore, immunofluorescence microscopy revealed prominent cytoplasmic colocalization of CCT2 and FASN in PCa cell lines (Figure S3A, Supporting Information). Consistently, proximity ligation assays (PLA) generated robust punctate signals, indicating a close molecular interaction between CCT2 and FASN in prostate cancer cells (Figure 3E).

Interestingly, Western blotting and qRT‐PCR analyses in PC‐3 and DU145 cells showed that CCT2 knockdown significantly decreased FASN protein abundance without altering FASN mRNA levels (Figure 3F and Figure S3B, Supporting Information), whereas CCT2 overexpression increased FASN protein abundance (Figure 3G and Figure S3C, Supporting Information). These findings suggested that CCT2 may regulate FASN in prostate cancer cells through post‐translational modification mechanisms. To further investigate this mechanism, we performed CHX chase assays and found that FASN protein half‐life was significantly shorter in cells subjected to CCT2 knockdown than in control cells (Figure 3H,J). Conversely, overexpressing CCT2 slowed FASN protein turnover (Figure 3I,K). Consistent with the observed effects on FASN turnover, treatment with the proteasome inhibitor MG132 largely rescued the reduction of FASN protein levels induced by CCT2 knockdown, indicating that CCT2‐dependent regulation of FASN stability involves a proteasome‐mediated degradation pathway (Figure 3L and Figure S3D, Supporting Information). Ubiquitination assays further revealed a marked increase in polyubiquitinated FASN upon CCT2 depletion, whereas re‐expression of siRNA‐resistant CCT2 significantly attenuated FASN ubiquitination, supporting a model in which CCT2 stabilizes FASN by limiting ubiquitin‐mediated proteasomal degradation (Figure 3M,N).

### 2.4 EIF3F Enhances FASN Protein Stability Through K48‐Linked Ubiquitin‒Proteasome‐Mediated Degradation

**FIGURE 4:**
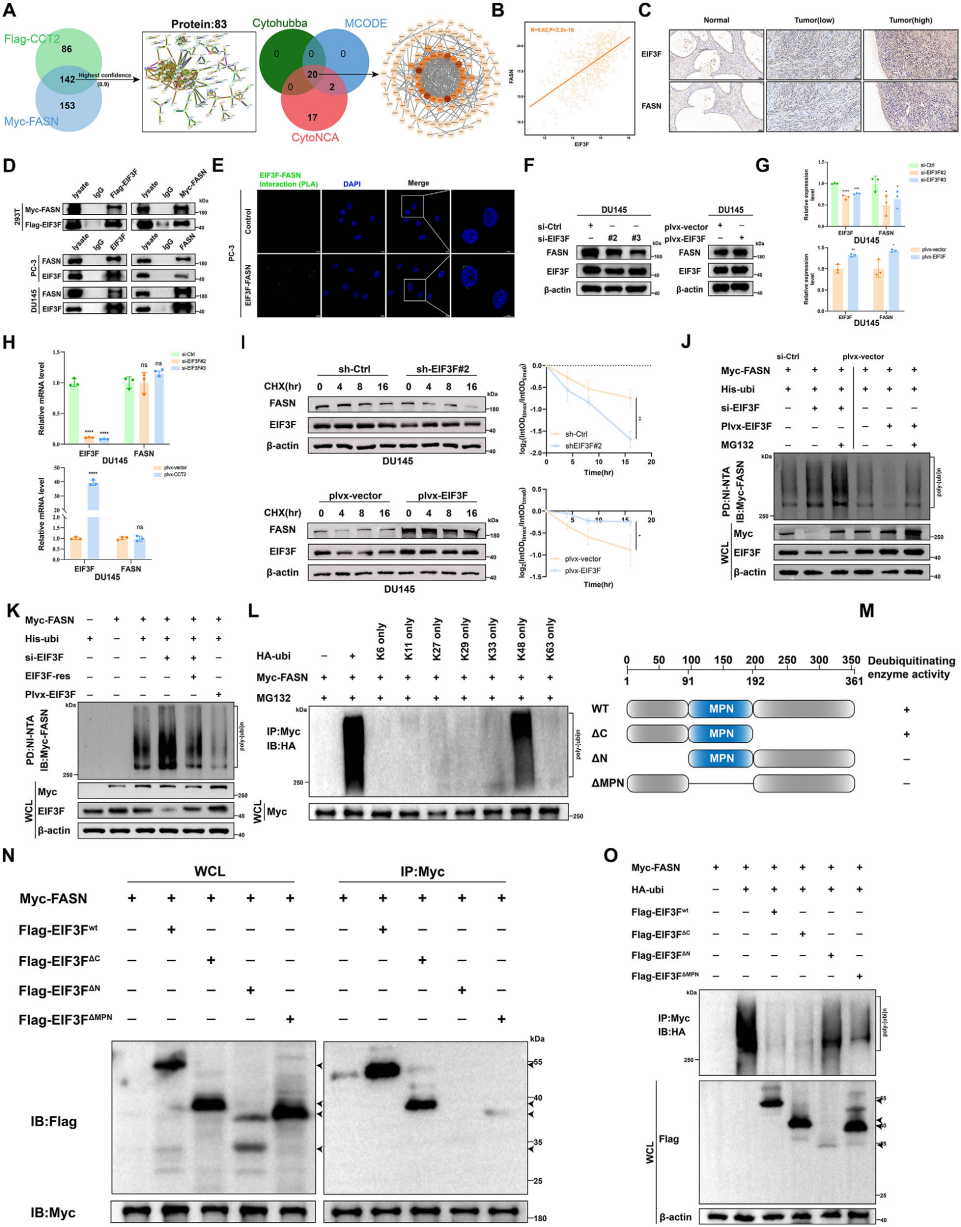
EIF3F modulates FASN protein stability through K48‐linked ubiquitin‐proteasome‐mediated degradation. (A) The intersection of IP‒MS data and network analyses (CytoHubba, MCODE, CytoNCA) highlights EIF3F as a core interactor. (B) Proteomic analysis of PCa patient samples was conducted to assess the correlation between EIF3F and FASN expression levels. Pearson's correlation coefficient (r) and corresponding *P* value are shown. (C)Representative immunohistochemical (IHC) staining images of EIF3F and FASN in normal prostate tissues and PCa tissues with low or high EIF3F expression. (D) Co‐IP assays were performed in HEK‐293T and PCa cells to validate the interaction between EIF3F and FASN. (E) Proximity ligation assay (PLA) showing in situ interaction between EIF3F and FASN in PC‐3 cells. Nuclei were counterstained with DAPI. (F‐H) qRT‐PCR and Western blotting analyses were used to assess the effects of EIF3F knockdown and overexpression on FASN mRNA and protein levels. Data are presented as mean ± SD from three independent biological replicates (n = 3). Statistical analysis was performed using one‐way ANOVA for EIF3F knockdown and two‐tailed unpaired Student's *t*‐tests for EIF3F overexpression. (I) CHX chase assays were used to evaluate the impact of EIF3F on FASN protein stability. Representative immunoblots and corresponding protein decay curves are shown. Protein band intensities were quantified by densitometry, normalized to β‐actin, and expressed relative to the 0 h time point. Decay constants (k) were obtained by linear regression of log_2_‐transformed normalized intensities. Data are presented as mean ± SD from three independent biological replicates (n = 3). Statistical significance was determined using a two‐tailed Student's *t*‐test. (J,K) Ni–NTA pull‐down assays detecting ubiquitinated FASN in cells transfected with His‐tagged ubiquitin, demonstrating that CCT2 suppresses FASN ubiquitination. (L) Ubiquitin linkage assays were conducted to determine the type of polyubiquitin chains modifying FASN. (M–O) Truncation and domain deletion analyses were performed to identify the EIF3F region required for its interaction with FASN and deubiquitination activity. For quantitative panels with significance annotations, ns indicates *p* > 0.05, *p < 0.05, **p < 0.01, ***p < 0.001, ****p < 0.0001.

Given the above experimental evidence for the CCT2‐mediated stabilization of FASN, we performed reciprocal IP‐MS using Flag‐tagged CCT2 and Myc‐tagged FASN in HEK‐293T cells to identify the potential regulatory mechanism. Proteomic analysis revealed 83 high‐confidence interacting proteins in the regulatory network. Comprehensive interaction mapping through the consensus of three network algorithms (CytoHubba, MCODE, and CytoNCA) revealed a 20‐protein core network (Figure 4A and Figure S4A,B, Supporting Information). Among these 20 proteins, only EIF3F has been previously reported to possess deubiquitinating activity [27]. In addition, previous proteomic analyses of PCa patient samples revealed a positive correlation between EIF3F and FASN expression [32], which we subsequently confirmed via IHC staining (Figure 4B,C). The EIF3F‐FASN interaction suggests a potential FASN deubiquitination mechanism that results in FASN stabilization.

Co‐IP assays confirmed the interaction between EIF3F and FASN in HEK‐293T cells and PCa cells (Figure 4D). IF and PLA analyses revealed predominant cytoplasmic colocalization and close spatial proximity of EIF3F and FASN in prostate cancer cells, supporting a specific intracellular interaction between the two proteins (Figure 4E and Figure S4C, Supporting Information). Similarly, EIF3F knockdown and overexpression significantly reduced and elevated FASN protein expression, respectively, while neither affected FASN mRNA levels (Figure 4F–H and Figure S4D,E, Supporting Information). Moreover, HPG‐based nascent protein labeling showed no significant changes in global protein synthesis upon EIF3F manipulation (Figure S4F,G, Supporting Information). CHX chase assays demonstrated that knockdown of EIF3F accelerated FASN protein turnover (Figure 4I and Figure S4H, Supporting Information), whereas overexpression had the opposite effect (Figure 4I and Figure S4H, Supporting Information). MG132 treatment attenuated the decrease in FASN protein levels caused by EIF3F knockdown and promoted the accumulation of ubiquitinated FASN, supporting a proteasome‐dependent degradation mechanism (Figure S4I, Supporting Information). Consistent with a proteasome‐dependent degradation mechanism, ubiquitination analyses revealed enhanced polyubiquitination of FASN upon EIF3F knockdown, which was effectively reversed by reintroduction of siRNA‐resistant EIF3F. This supports a regulatory role for EIF3F in maintaining FASN protein stability through suppression of ubiquitin‐mediated proteasomal turnover (Figure 4J,K). Ubiquitin linkage analysis demonstrated that FASN is predominantly modified by K48‐linked polyubiquitin chains rather than K63‐linked chains (Figure 4L). Given that K48‐linked ubiquitination typically targets proteins for proteasomal degradation, this result indicates that FASN stability is primarily regulated through degradation‐dependent mechanisms. Both MPN domain deletion (ΔMPN) and N‐terminal truncation (ΔN) significantly impaired the deubiquitination activity and FASN binding of EIF3F, with the MPN domain playing a predominant role in mediating this interaction and catalytic function (Figure 4M–O). Overall, these findings show that EIF3F stabilizes FASN by deubiquitinating it and inhibiting its proteasomal degradation.

### 2.5 CCT2 Functions as a Ternary Complex Stabilizer That Potentiates the EIF3F‐FASN Interaction, Leading to Increased FASN Deubiquitination

**FIGURE 5:**
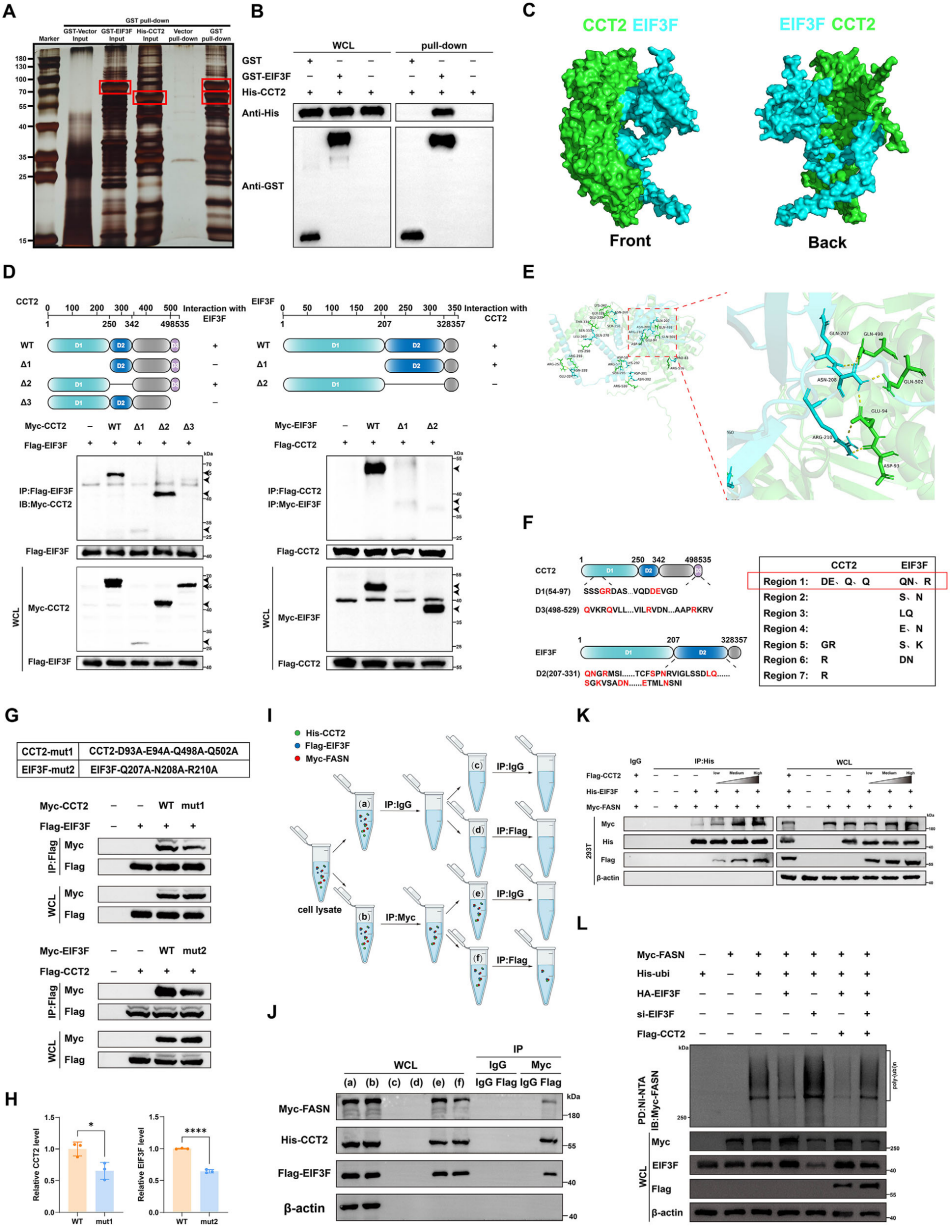
CCT2 facilitates FASN stabilization by enhancing the EIF3F‐FASN interaction via ternary complex formation. (A) Silver staining of purified His‐CCT2 and GST‐EIF3F recombinant proteins. (B) GST pull‐down assays were performed to assess the direct in vitro interaction between CCT2 and EIF3F. (C) Predicted 3D structural models of CCT2 and EIF3F, with docking simulations suggesting multiple potential interaction regions. (D) A schematic of the EIF3F and CCT2 truncation constructs and subsequent Co‐IP assays using domain‐specific mutants identified CCT2‐D1/3 and EIF3F‐D2 as the principal interaction surfaces. (E, F) High‐resolution molecular docking of Region 1 showing a stable binding conformation with critical amino acid residues (DEQQ in CCT2 and QNR in EIF3F) contributing to the interaction interface. (G‐H) Co‐IP assays using point mutants (CCT2‐mut1 and EIF3F‐mut2) were conducted to assess the functional importance of Region 1. Data are presented as mean ± SD from three independent biological replicates (n = 3). Statistical significance was assessed using two‐tailed unpaired Student's *t*‐tests. (I,J) Schematic diagrams and Co‐IP data illustrate the formation of a trimeric complex comprising CCT2, EIF3F, and FASN. (K) Co‐transfection of His‐EIF3F and Myc‐FASN with increasing doses of Flag‐CCT2 in HEK‐293T cells, followed by Western blot analysis to assess the impact of CCT2 levels on EIF3F‐mediated FASN complex formation. (L) Denaturing Ni‐NTA pull‐down assays showing that CCT2 enhances EIF3F‐mediated deubiquitination of FASN in the presence of His‐ubiquitin. For quantitative panels with significance annotations, *p < 0.05, ****p < 0.0001.

To elucidate the role of CCT2 in regulating the EIF3F‐FASN interaction, both endogenous and exogenous Co‐IP assays were performed, which revealed a physical interaction between CCT2 and EIF3F (Figure S6A, Supporting Information); this interaction was further validated by IF staining and PLA assay (Figure S6B,C, Supporting Information). Next, GST pull‐down assays were conducted using purified GST‐tagged EIF3F and His‐tagged CCT2 fusion proteins. The results demonstrated a direct interaction between CCT2 and EIF3F (Figure 5A,B and Figure S6D, Supporting Information). Computational modeling, including protein structure prediction and molecular docking, was used to generate three‐dimensional models of CCT2 and EIF3F and to predict their potential interaction interfaces, providing structural insights into their functional association (Figure 5C). From these predicted structures, seven potential binding regions between the two proteins were identified (Figure S6E,F, Supporting Information). We then constructed various truncation mutants of CCT2 and EIF3F on the basis of the predicted interaction sites. Co‐IP assays revealed that CCT2‐D1, CCT2‐D3, and EIF3F‐D2 were the regions with the greatest impact on CCT2‐EIF3F binding (Figure 5D). Among the seven predicted binding regions, the experimental findings were consistent with binding in Regions 1, 5, or 6. However, considering both the three‐dimensional structural model and the binding affinity experiment results, Region 1 was identified as the most likely interaction site (Figure 5E,F). To further validate Region 1 as the key interaction interface, corresponding mutants (CCT2‐mut1 and EIF3F‐mut2) targeting this region were generated and used in subsequent co‐immunoprecipitation assays to assess binding specificity, as well as in preliminary screening for potential small molecules that may disrupt this interaction. The results indicated that the amino acid residues within Region 1 play a critical role in mediating the interaction between CCT2 and EIF3F (Figure 5G,H). Importantly, loss‐ and gain‐of‐function analyses showed that altering CCT2 expression did not affect EIF3F protein abundance or stability, nor did EIF3F perturbation influence CCT2 expression or turnover, as determined by immunoblotting and CHX chase assays in PC‐3 and DU145 cells (Figure S5A–L, Supporting Information). These findings indicate that CCT2 and EIF3F do not regulate each other at the level of protein stability, supporting a model in which CCT2 primarily acts as a structural scaffold.

Subsequently, a series of immunoprecipitation experiments were conducted, and the His‐CCT2 signal was successfully detected (Figure 5I,J), further confirming that CCT2, EIF3F, and FASN are components of the same protein complex. CCT2 enhanced the interaction between EIF3F and FASN in a dose‐dependent manner and promoted EIF3F‐mediated deubiquitination of FASN (Figure 5K,L and Figure S6G, Supporting Information). CCT2 suppressed K48‐linked polyubiquitination of FASN, an effect dependent on EIF3F but not USP2 or TRIM21 (Figure S6H, Supporting Information), indicating that CCT2 stabilizes FASN by facilitating EIF3F‐dependent proteasomal protection.

### 2.6 The Small Molecule Y043‐8015 Suppresses PCa Progression by Targeting the CCT2‐EIF3F‐FASN Complex

**FIGURE 6:**
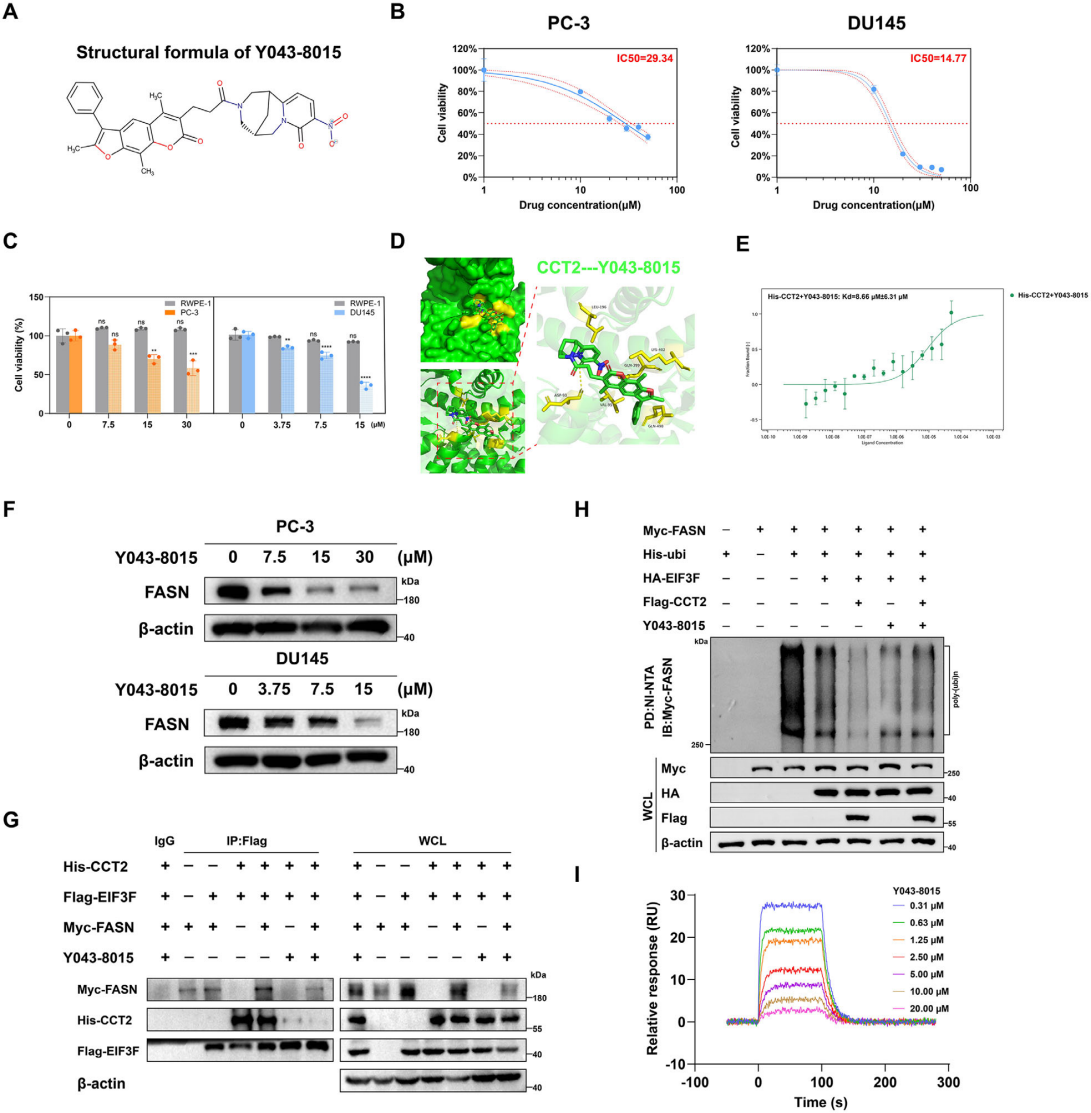
Y043‐8015 suppresses PCa progression by disrupting the CCT2‐EIF3F‐FASN complex. (A) Chemical structure of Y043‐8015. (B) Dose‐response curves showing the IC_50_ values of Y043‐8015 in PC‐3 and DU145 PCa cells. (C) CCK‐8 assays showing that Y043‐8015 selectively inhibits the viability of prostate cancer cells (PC‐3 and DU145) in a dose‐dependent manner, while exerting minimal cytotoxic effects on normal prostate epithelial cells (RWPE‐1) at the indicated concentrations. Data are presented as mean ± SD from three independent biological replicates (n = 3). Statistical significance was assessed using one‐way ANOVA. (D) Molecular docking images showing predicted binding of Y043‐8015 to the surface of the CCT2 protein. (E) MST analysis showing the binding affinity between recombinant His‐tagged CCT2 protein and compound Y043‐8015. Fluorescence changes were monitored across a range of ligand concentrations to generate a binding curve and determine the dissociation constant (Kd). (F) Western blotting was performed to assess FASN protein levels following treatment with dose‐dependent concentrations of Y043‐8015. (G) Co‐IP assays were used to evaluate the effect of Y043‐8015 on the interaction between CCT2 and EIF3F. (H) Denaturing Ni‐NTA ubiquitination assays demonstrating that Y043‐8015 increases FASN polyubiquitination by interfering with the CCT2–EIF3F–FASN regulatory axis. (I) Direct competition assay showing that Y043‐8015 weakens the binding between CCT2 and EIF3F in a dose‐dependent manner. For quantitative panels with significance annotations, ns indicates *p* > 0.05, **p < 0.01***p < 0.001****p < 0.0001.

Our findings above established the critical role of the CCT2‐EIF3F‐FASN complex in PCa, suggesting that the development of specific inhibitors targeting this complex could offer a novel therapeutic strategy. To identify such inhibitors, we performed molecular docking‐based virtual screening of approximately 150 000 small‐molecule compounds from the Bioactive and Drugs subset of the ZINC database using the crystal structure of Region 1 of the CCT2 protein, which yielded nine candidate compounds. The effects of these compounds on PC‐3 and DU145 cell proliferation were evaluated through CCK‐8 assays, and Y043‐8015 exhibited the most potent inhibitory effects (Figure 6A,B and Figure S7A,B, Supporting Information). Notably, Y043‐8015 significantly suppressed the viability of PCa cells while exerting minimal effects on normal prostate epithelial cells (Figure 6C), suggesting favourable safety and promising therapeutic potential. Molecular docking revealed a stable binding conformation between Y043‐8015 and the region 1 interface of CCT2 (Figure 6D). Microscale thermophoresis (MST) further confirmed the interaction, with the fraction bound of His‐CCT2 increasing from 0 to nearly 1 in a dose‐dependent manner. The dissociation constant (Kd) was determined to be 8.66 ± 6.31 µM, indicating a specific binding affinity between His‐CCT2 and Y043‐8015 (Figure 6E and Figure S7C).

In PCa cells, Y043‐8015 reduced FASN protein expression in a dose‐dependent manner (Figure 6F). Mechanistically, Y043‐8015 significantly disrupted the interaction between CCT2 and EIF3F, thereby attenuating the deubiquitination activity of EIF3F on FASN. This led to decreased FASN protein stability and enhanced proteasomal degradation (Figure 6G,H). Importantly, direct competition analysis demonstrated that Y043‐8015 attenuated CCT2–EIF3F binding in a concentration‐dependent manner, providing direct support that the compound targets this interaction interface (Figure 6I). Collectively, these findings suggest that Y043‐8015 disrupts the CCT2‐EIF3F‐FASN complex, thereby inhibiting PCa cell proliferation and highlighting its potential as a therapeutic agent.

### 2.7 FOXA1 Transcriptionally Activates CCT2 by Binding to its Promoter Region

**FIGURE 7:**
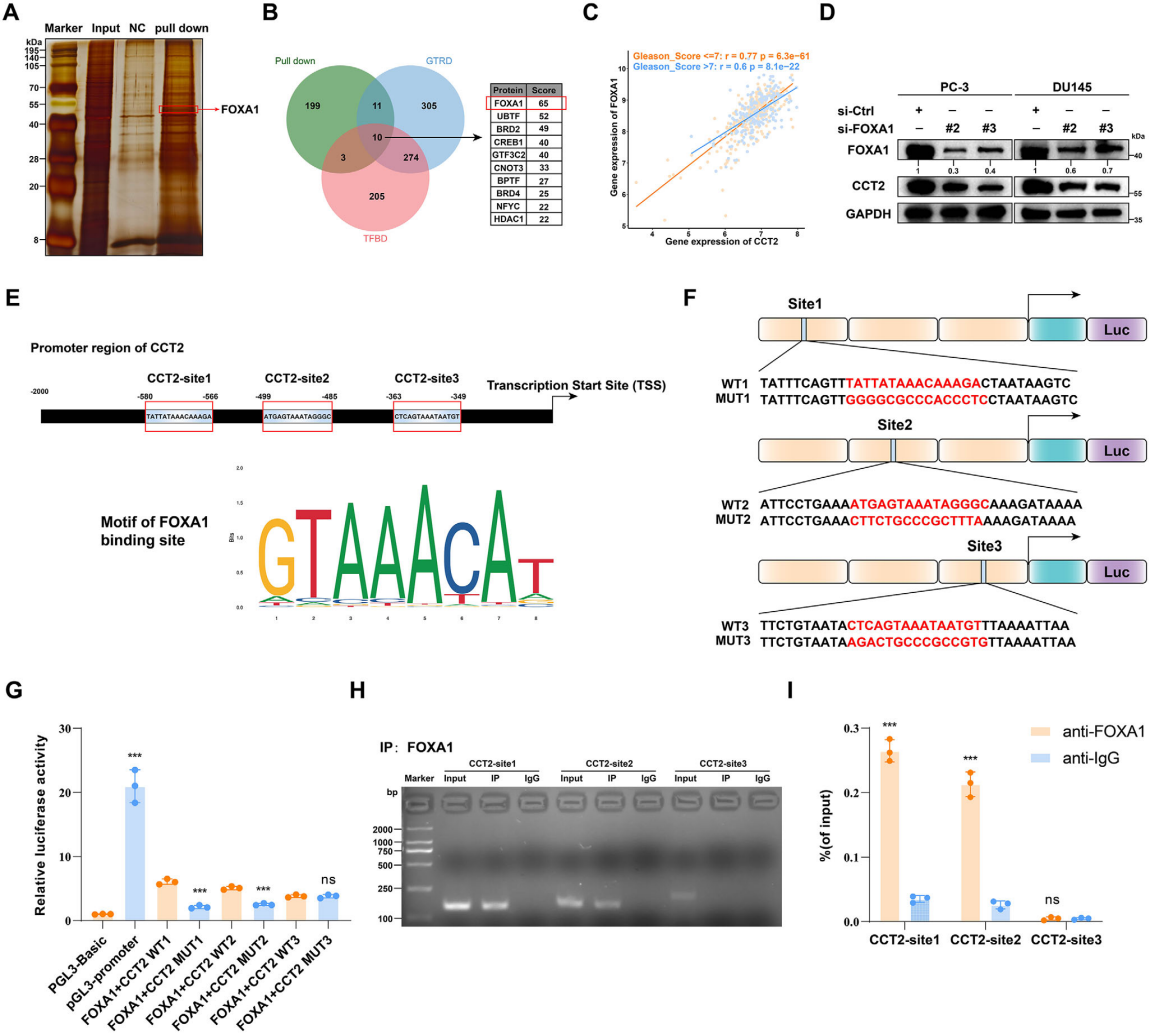
FOXA1 directly regulates CCT2 transcription through promoter binding. (A) Silver staining of DNA pull‐down products. (B) Venn diagram of transcription factor candidates identified by DNA pull‐down, TFBD, and GTRD analyses highlighting FOXA1. (C) Correlation analysis of TCGA data showing the relationship between FOXA1 and CCT2 expression. (D) Western blotting was used to assess CCT2 expression following FOXA1 knockdown. (E) FOXA1 binding sites and consensus motif in the CCT2 promoter. (F) Schematic of wild‐type and mutant CCT2 promoter luciferase constructs. (G) Dual‐luciferase reporter assays were performed to evaluate the effect of FOXA1 on CCT2 promoter activity and the functional relevance of predicted binding sites. Data are presented as mean ± SD from three independent biological replicates (n = 3). Statistical significance was assessed using one‐way ANOVA. (H,I) ChIP‐PCR confirmed FOXA1 occupancy at the CCT2 promoter in PCa cells. Data are presented as mean ± SD from three independent biological replicates (n = 3). Statistical significance was assessed using two‐tailed unpaired Student's *t*‐test. For quantitative panels with significance annotations, ns indicates *p* > 0.05, ***p < 0.001.

To investigate the upstream regulatory mechanism of CCT2, we compared the results of the DNA pull‐down assay with those of transcription factor predictions from the GTRD and TFBD databases. FOXA1 had the highest score (Figure 7A,B). Correlation analysis based on the TCGA dataset revealed a positive association between FOXA1 and CCT2 expression levels (Figure 7C and Figure S8A, Supporting Information), which was further validated by IHC staining (Figure S8B, Supporting Information). To further elucidate the mechanism by which FOXA1 regulates CCT2, we performed FOXA1 knockdown, which led to a marked decrease in CCT2 expression (Figure 7D and Figure S8C,D, Supporting Information). In contrast, depletion of FOXA1 did not significantly alter EIF3F or FASN mRNA expression, nor did it affect their protein abundance in either cell line (Figure S8E–G, Supporting Information), indicating that FOXA1 selectively regulates CCT2 transcription rather than directly controlling EIF3F or FASN expression. Using the JASPAR database, we predicted potential FOXA1‐binding sites within the CCT2 promoter region and subsequently constructed CCT2 wild‐type (WT) and mutant (MUT) reporter plasmids (Figure 7E,F). Luciferase reporter assays demonstrated that FOXA1 significantly enhanced the promoter activity of CCT2‐WT, whereas this effect was markedly diminished in the CCT2‐MUT1 and MUT2 constructs (Figure 7G). Consistently, ChIP assays confirmed that FOXA1 predominantly binds to site 1 and site 2 within the CCT2 promoter (Figure 7H,I). Together, these findings indicate that FOXA1 activates CCT2 transcription by directly binding to its promoter region.

### 2.8 Pharmacological Inhibition of FASN or Disruption of CCT2‐EIF3F Interaction Attenuates Tumor Growth and Metastatic Burden In Vivo

To evaluate the effects of orlistat and Y043‐8015 on the malignant phenotypes of prostate cancer cells, PC‐3 and DU145 cells with normal or enforced CCT2 expression were treated with orlistat, Y043‐8015, or their combination. Colony formation assays demonstrated that either orlistat or Y043‐8015 alone significantly suppressed prostate cancer cell growth, whereas combined treatment exerted the most pronounced inhibitory effect, particularly in CCT2‐overexpressing cells (Figure S9A, Supporting Information). Consistently, wound‐healing and transwell assays revealed that the increased migratory and invasive capacities associated with CCT2 overexpression were attenuated by orlistat or Y043‐8015, with the strongest suppression observed upon combined treatment (Figure S9B,C, Supporting Information). In addition, Oil Red O staining showed that CCT2 overexpression markedly promoted intracellular lipid droplet accumulation, which was substantially reduced following treatment with orlistat or Y043‐8015 (Figure S9D, Supporting Information). Furthermore, to explore the role of the CCT2/EIF3F/FASN axis in EMT regulation, we first confirmed endogenous interactions among CCT2, EIF3F, and FASN in RM‐1 cells by co‐immunoprecipitation (Figure S10A, Supporting Information). Functionally, CCT2 overexpression increased FASN protein levels, whereas EIF3F knockdown significantly attenuated this effect (Figure S10B,C, Supporting Information), indicating that CCT2‐mediated FASN stabilization depends on EIF3F. Consistently, ubiquitination assays showed that CCT2 reduced FASN polyubiquitination, which was largely reversed upon EIF3F depletion (Figure S10D, Supporting Information). Moreover, CCT2 overexpression promoted EMT‐associated changes, characterized by increased Vimentin, N‐cadherin, SLUG, and SNAI1 expression and reduced E‐cadherin levels. Importantly, these EMT phenotypes were markedly suppressed by EIF3F knockdown or pharmacological inhibition of FASN using Orlistat or Y043‐8015 (Figure S10E,F, Supporting Information). In line with these observations, both treatments also markedly impaired the migratory and invasive capacities of RM‐1 cells (Figure S10G,H, Supporting Information). Together, these data are consistent with a model in which the CCT2/EIF3F/FASN axis is associated with EMT‐related phenotypic alterations.

**FIGURE 8:**
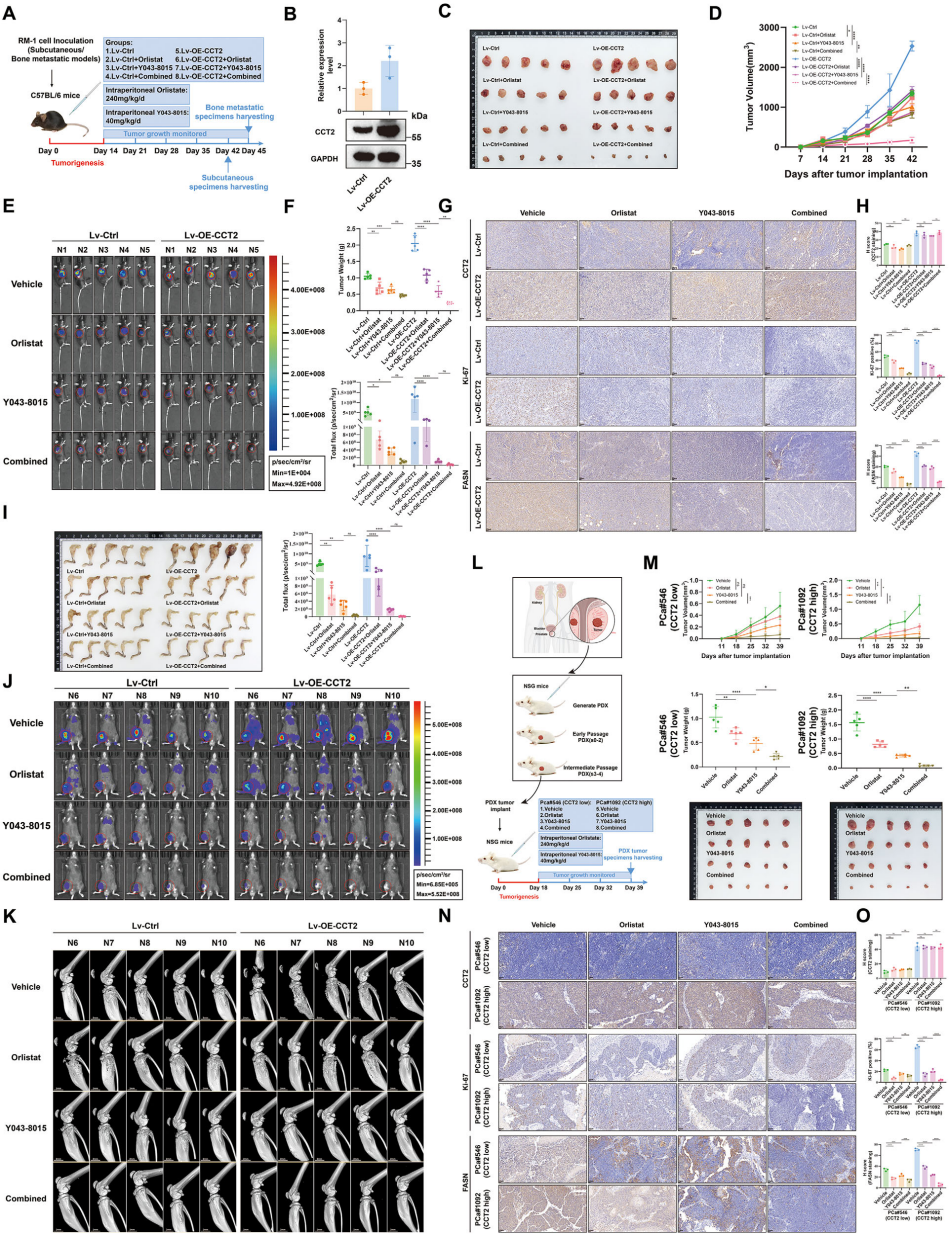
Dual targeting of the CCT2 metabolic axis by Orlistat and Y0438015 reduces tumor growth and bone metastatic burden in multiple in vivo prostate cancer models. (A) Schematic overview of the subcutaneous and bone metastasis tumor models established in C57BL/6 mice. (B) Western blotting analysis confirming CCT2 overexpression in RM‐1 cells. Data are presented as mean ± SD from three independent biological replicates (n = 3). Statistical significance was assessed using two‐tailed unpaired Student's *t*‐test. (C) Representative images of subcutaneous tumors. (D) Tumor growth curves of subcutaneous isografts treated with Orlistat, Y043‐8015, or their combination. Sample size was n = 5 mice per group. Statistical significance was assessed using two‐way ANOVA. (E) Bioluminescence imaging of bone metastatic lesions after intratibial injection of RM‐1 cells and quantification of bioluminescence signals from panel. (F) Quantification of subcutaneous tumor weight at endpoint across treatment groups. Data are presented as mean ± SD (n = 5 mice per group). Statistical significance was assessed using one‐way ANOVA within each genetic background (Lv‐Ctrl or Lv‐OE‐CCT2). (G) Representative IHC staining of CCT2, Ki‐67, and FASN in subcutaneous tumor sections across different treatment groups. (H) Quantification of IHC staining intensity using H‐score. Data are presented as mean ± SD (n = 5 mice per group). Statistical significance was assessed using one‐way ANOVA within each genetic background (Lv‐Ctrl or Lv‐OE‐CCT2). (I) Representative images of ex vivo bone tissues collected from bone metastasis models. (J) Bioluminescence imaging of RM‐1 bone metastases in C57BL/6 mice established by intratibial injection and quantification of total bioluminescence flux. Data are presented as mean ± SD (n = 5 mice per group). Statistical significance was assessed using one‐way ANOVA within each genetic background (Lv‐Ctrl or Lv‐OE‐CCT2). (K) Micro‐CT images showing representative osteolytic changes in RM‐1 metastatic models. (L) Schematic diagram illustrating the establishment of a patient‐derived xenograft (PDX) model of prostate cancer in NSG mice. (M) Tumor growth curves and endpoint tumor weights, and representative tumor images from subcutaneous xenografts. Data are presented as mean ± SD (n = 5 mice per group). Statistical significance for tumor growth curves was assessed using two‐way ANOVA, and endpoint tumor weights were analyzed using one‐way ANOVA within each PDX model. (N) IHC staining of CCT2, FASN, and Ki‐67 in PDX tumors treated with Orlistat, Y043‐8015, or their combination. (O) Quantitative analysis of IHC staining. Data are presented as mean ± SD (n = 5 mice per group). Statistical significance was assessed using one‐way ANOVA within each PDX model. For quantitative panels with significance annotations, ns indicates *p* > 0.05, *p < 0.05, **p < 0.01, ***p < 0.001, ****p < 0.0001.

Before initiating in vivo therapeutic studies, we performed a dose‐escalation pilot experiment to determine the optimal effective concentration of Y043‐8015. Subcutaneous RM‐1 tumor–bearing mice were treated with increasing doses of Y043‐8015 (10‐60 mg/kg), and tumor burden assessment revealed that 40 mg/kg achieved the most robust antitumor efficacy. Importantly, mice treated with Y043‐8015 at 40 mg/kg did not exhibit obvious body weight loss during the treatment period, and H&E staining of major organs, including the heart, liver, spleen, lung, and kidney, showed no apparent histopathological abnormalities compared with vehicle‐treated controls. These findings support that 40 mg/kg was well tolerated in vivo and was therefore selected for subsequent in vivo experiments (Figure S10I–K, Supporting Information). To further evaluate the therapeutic potential of targeting CCT2‐mediated metabolic pathways in vivo, multiple prostate cancer mouse models were established under both immunocompetent and immunodeficient conditions. RM‐1 cells were implanted subcutaneously or injected intra‐tibially into C57BL/6 mice to generate subcutaneous tumor and bone metastasis models, respectively (Figure 8A). Successful CCT2 overexpression was confirmed by Western blotting (Figure 8B). In vivo tumorigenicity assays showed that CCT2 overexpression (Lv‐OE‐CCT2) markedly accelerated tumor growth. In contrast, treatment with orlistat or Y043‐8015 alone significantly suppressed tumor growth, while combined treatment exerted a more pronounced inhibitory effect (Figure 8C,I). Quantitative analyses of tumor volume and tumor weight further corroborated these observations (Figure 8D,F). Moreover, bioluminescence imaging revealed that both orlistat and Y043‐8015 treatments significantly reduced tumor burden and decreased the incidence of bone metastatic lesions in the bone colonization model, with more prominent effects observed in the context of CCT2 overexpression (Figure 8E,J). Consistently, micro‐computed tomography (micro‐CT) analysis demonstrated that mice receiving combined treatment exhibited markedly reduced osteolytic lesions and preserved bone architecture, supporting the inhibitory effects of dual treatment on metastatic burden (Figure 8K and Figure S10L, Supporting Information). Histological analyses of subcutaneous tumors and bone metastatic lesions further showed that treatment with orlistat or Y043‐8015, either alone or in combination, significantly reduced tumor cell proliferation, as evidenced by decreased Ki‐67 staining. In parallel, lipid metabolic activity within tumor tissues was markedly suppressed, as reflected by reduced FASN expression. In addition, the expression of the mesenchymal marker N‐cadherin was substantially decreased, whereas the epithelial marker E‐cadherin was restored, indicating attenuation of the epithelial–mesenchymal transition (EMT) phenotype. Notably, these effects were most pronounced in CCT2‐overexpressing tumors (Figure 8G,H and Figure S10M, Supporting Information). Given the observed association between CCT2 expression and metastatic burden in vivo, we further analyzed clinical prostate cancer datasets from the SU2C/PCF cohort. Comparative analysis revealed that CCT2 expression was significantly higher in prostate cancer patients with bone metastases than in those with primary prostate tumors alone (Figure S10N, Supporting Information), supporting the clinical relevance of CCT2 in prostate cancer bone metastasis.

Subsequently, prostate cancer patient‐derived xenograft (PDX) models were established in immunodeficient NSG mice to evaluate the antitumor effects of orlistat and Y043‐8015 on tumor growth (Figure 8L). In two PCa‐PDX cohorts stratified by CCT2 expression (CCT2‐high versus CCT2‐low), PDX tumors with high CCT2 expression exhibited greater sensitivity to pharmacological intervention: combined treatment with orlistat and Y043‐8015 suppressed tumor growth more effectively than either agent alone. In contrast, the antitumor efficacy of these treatments was markedly attenuated in CCT2‐low PDX tumors, with only limited reductions observed in tumor volume and tumor weight (Figure 8M).

Further immunohistochemical (IHC) analyses demonstrated that, in the CCT2‐high group, combined treatment significantly reduced the expression of Ki‐67 and FASN, whereas these effects were comparatively modest in CCT2‐low tumors (Figure 8N,O). Collectively, these findings indicate that combined treatment exerts a more potent inhibitory effect on tumor growth and associated molecular markers in the context of high CCT2 expression.

## 3 Discussion

PCa progression is increasingly linked to dysregulated lipid metabolism, but the upstream molecular mechanisms that control this metabolic reprogramming remain poorly understood. In this study, we sought to uncover novel regulators of lipid metabolism in PCa by integrating transcriptomic, proteomic, and functional screening approaches. Through this unbiased analysis, we identified CCT2 as a key regulator. CCT2 functions as both a chaperone protein and an autophagy receptor [33, 34], and has been shown to promote the progression of various malignancies, including colorectal, ovarian, and breast cancers [9, 10, 11]. Lipid synthesis is closely associated with PCa progression [35, 36, 37, 38]; however, until this work, the regulatory role of CCT2 in lipid metabolism in PCa had not been explored. Here, we identified CCT2 as a key regulator of lipogenesis that enhances tumor cell proliferation and motility by promoting fatty acid synthesis and lipid droplet formation, thereby contributing to PCa progression. We further showed that CCT2 facilitates the interaction between EIF3F and FASN, the rate‐limiting enzyme in the de novo fatty acid synthesis pathway, promoting the EIF3F‐mediated deubiquitination of FASN and thereby enhancing its stability. Accordingly, the tumor‐promoting effects of CCT2 on prostate cancer growth and metastatic phenotypes were effectively mitigated by pharmacological intervention. In addition to FASN inhibition by orlistat, disruption of the CCT2‐EIF3F interaction using the small‐molecule inhibitor Y043‐8015 also significantly suppressed tumor progression. Notably, combined treatment with orlistat and Y043‐8015 exerted a more pronounced antitumor effect than either agent alone, particularly in tumors with elevated CCT2 expression, highlighting the therapeutic advantage of simultaneously targeting lipid metabolism and CCT2‐dependent protein stabilization pathways. Moreover, we showed that CCT2 transcription is directly activated by FOXA1, which was previously recognized as a key transcription factor in prostate cancer [39, 40] through an unknown mechanism. In this study, we demonstrate that FOXA1 directly activates CCT2 transcription and, more importantly, uncover a previously uncharacterized oncogenic pathway whereby CCT2 modulates the EIF3F‐FASN ubiquitin‐dependent lipid metabolic axis to promote tumor cell proliferation and metastasis.

A key finding of our study is that FASN stability in PCa cells is regulated by its interaction with the CCT2‐EIF3F complex, which inhibits its polyubiquitination and subsequent proteasomal degradation. FASN is a key enzyme in lipid synthesis, and its dysregulation is closely associated with tumor development. At the transcriptional level, the architectural transcription factor high mobility group A1 (HMGA1) enhances the transcriptional activity of SREBP1 at the FASN promoter, thereby upregulating FASN expression. This regulatory mechanism promotes lipid accumulation in intestinal epithelial cells and contributes to the progression of rectal cancer [41]. Along with transcriptional regulation, post‐translational modifications play a critical role in modulating FASN activity. APOE promotes the malignant progression of pancreatic neuroendocrine tumors by regulating FASN ubiquitination, thereby activating the PI3K/AKT/mTOR signaling pathway [42]. Similarly, recent studies in colorectal cancer have shown that FABP5 and CBR4 both promote ubiquitin–proteasome‐mediated degradation of FASN, thereby suppressing FASN/mTOR signaling, lipid accumulation, and tumor progression [43, 44] In liver cancer, FASN palmitoylation is mediated by zinc finger DHHC‐type palmitoyltransferase 20 (ZDHHC20), which competes with SNX8‐TRIM28‐mediated ubiquitination. This interplay between palmitoylation and ubiquitination facilitates FASN stabilization and contributes to tumor initiation and progression [45]. Previous studies have suggested that the synergistic interaction between fatty acids and cholesterol may play a critical role in the progression of prostate intraepithelial neoplasia (PIN) to invasive carcinoma [38]. FASN, a central enzyme in lipid metabolism, is commonly overexpressed in PCa and markedly enhances fatty acid synthesis in tumor cells [46, 47]. Collectively, these findings support an important role for FASN in PCa progression. In PCa, upstream FASN regulation is primarily mediated at the transcriptional level by SREBP1 and c‐Myc [35, 48]. The recently developed antitumor drug darolutamide suppresses FASN expression through this mechanism by downregulating SREBP1, thereby disrupting phospholipid remodeling, inducing ferroptosis, and inhibiting tumor progression [49]. *Eriobotrya japonica* extract (EJCE) has demonstrated comparable regulatory effects in PCa treatment [37]. However, the post‐translational regulation of FASN in PCa has been less explored. In this study, we found that CCT2 does not affect FASN mRNA levels but rather contributes to PCa progression by facilitating the interaction between FASN and the deubiquitinase EIF3F. This interaction attenuates K48‐linked ubiquitination and proteasomal degradation of FASN, thereby increasing its protein stability and expression. These findings expand the current understanding of lipid metabolism regulation by revealing a ubiquitin‐dependent mechanism. A deeper investigation into the post‐translational regulatory mechanisms of FASN may not only elucidate the metabolic reprogramming underlying PCa progression but also provide a rationale for therapeutic exploration.

As a core subunit of the EIF3 complex, EIF3F has been implicated not only in mRNA translation but also in key oncogenic processes including cell proliferation, apoptosis, and metabolic reprogramming. Recent studies have revealed that EIF3F possesses notable deubiquitinase activity and contributes to the progression of various malignancies by specifically regulating the stability of distinct substrate proteins [27]. In colorectal cancer, EIF3F has been reported to promote tumor progression by antagonizing the ubiquitination and degradation of PHGDH [27]. In hepatocellular carcinoma, EIF3F specifically deubiquitinates and stabilizes acyl‐CoA synthetase long‐chain family member 4 (ACSL4) through K48‐linked deubiquitination, thereby increasing fatty acid biosynthesis and accelerating tumor progression [50]. The substrate selectivity of EIF3F may be influenced by the tissue‐specific microenvironment or unique molecular characteristics of each tumor. Previous studies have demonstrated that EIF3F is highly expressed in PCa and promotes tumor growth by activating signaling pathways such as the Akt pathway [28], suggesting that EIF3F may employ a similar regulatory mechanism in PCa. Our study revealed that EIF3F promotes lipid synthesis and malignant progression in PCa cells by interacting with both CCT2 and FASN, thereby facilitating the stabilization of FASN through inhibition of its ubiquitination and degradation. This finding aligns with the established oncogenic role of EIF3F and highlights its noncanonical function within the ubiquitin–proteasome system. Notably, these findings underscore the key regulatory role of EIF3F in the lipid metabolism network of PCa, offering new insights into the mechanisms underlying EIF3F‐associated tumor progression. Targeting the CCT2–EIF3F–FASN axis, particularly through the identification of small‐molecule compounds that disrupt the CCT2–EIF3F interaction (such as CCT2 inhibitors), may selectively impair lipid metabolism in PCa while preserving the translational regulatory functions of EIF3F. This study focused primarily on the role of EIF3F in lipid metabolism; however, its functional alterations in protein translation regulation remain to be fully elucidated. Moreover, the expression patterns of EIF3F across different PCa subtypes and their clinical implications warrant further investigation.

The identification of the CCT2‐EIF3F‐FASN axis as an important regulatory axis of lipid metabolism in PCa provides mechanistic rationale for the development of novel targeted therapies. Through high‐throughput screening, we identified Y043‐8015 as a specific compound that binds to CCT2 and competitively disrupts its interaction with EIF3F, thereby suggesting a mechanistic link between chaperone‐translation factor crosstalk and metabolic vulnerability in prostate cancer. Importantly, the selective cytotoxicity observed in PCa cells versus normal prostate epithelial cells suggests potential selectivity. These findings extend previous efforts targeting lipid metabolism in cancer by demonstrating that protein‐protein interactions upstream of FASN can be effectively modulated. As pharmacologic inhibitors of metabolic enzymes often suffer from compensatory resistance, targeting scaffold‐like interactions such as CCT2‐EIF3F may represent a complementary therapeutic strategy. These findings highlight the potential of Y043‐8015 as a promising therapeutic agent. Further studies are warranted to evaluate its synergistic effects with existing treatments, such as enzalutamide, in PCa therapy.

Despite the strengths of our study, several limitations should be acknowledged. First, Single‐cell RNA sequencing revealed that CCT2 is highly expressed not only in PCa epithelial cells but also in tumor‐infiltrating T cells and vascular endothelial cells. This expression pattern suggests that CCT2 may contribute to tumor progression through synergistic functions in multiple cell types, warranting further investigation in future studies. Second, although Y043‐8015 showed promising antitumor effects and selectivity, its pharmacokinetics, toxicity, and off‐target profiles remain to be fully evaluated in preclinical models.

**FIGURE 9:**
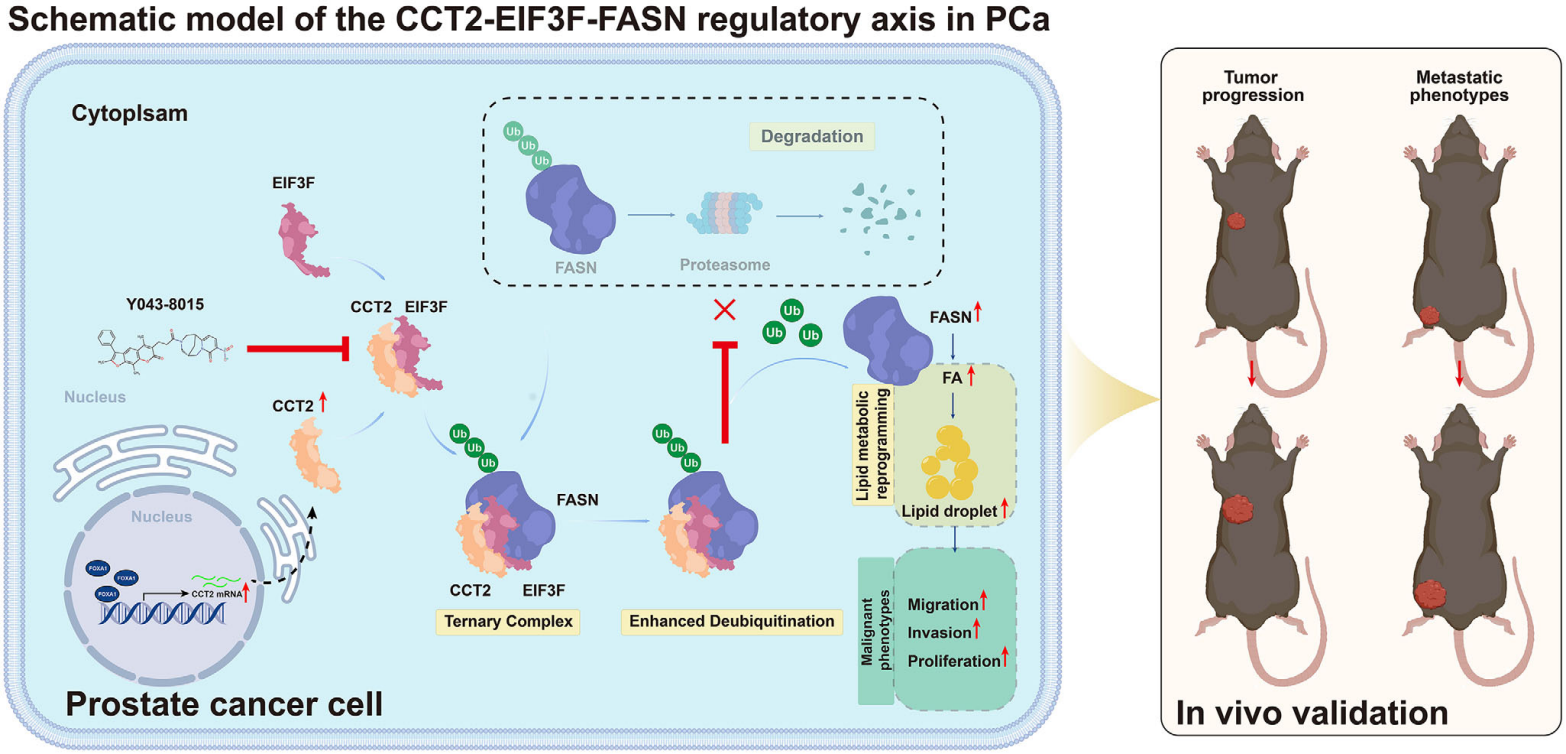
Schematic model illustrating the role of the FOXA1‐CCT2‐EIF3F‐FASN axis in regulating lipid metabolic reprogramming and associated malignant phenotypes in PCa.

In summary, this study provides mechanistic insight into the regulatory mechanism by which FOXA1 transcriptionally upregulates CCT2, thereby facilitating EIF3F‐mediated deubiquitination and stabilization of FASN, ultimately contributing to lipid metabolic reprogramming in PCa (Figure 9). This discovery not only enriches our understanding of the oncogenic network associated with FOXA1 but also extends the functional scope of CCT2 as a molecular chaperone to include the regulation of ubiquitin‐dependent lipid metabolism in PCa. Importantly, pharmacological targeting of this pathway using the FASN inhibitor orlistat and the CCT2‐EIF3F interaction disruptor Y043‐8015 effectively suppressed tumor growth and metastatic phenotypes both in vitro and in vivo. Notably, combined treatment produced a more pronounced antitumor effect than either agent alone, particularly in CCT2‐overexpressing tumors, supporting the rationale for further exploration of concurrently targeting lipid metabolism and CCT2‐dependent protein stabilization. Future research will focus on elucidating how CCT2 may contribute to PCa progression through multicellular crosstalk, which could enhance our understanding of the metabolic‐immune regulatory axis in the tumor microenvironment. In parallel, efforts will be directed toward developing precision therapeutic strategies that leverage these interactions.

## 4 Experimental Section

*Gene expression profiles, clinical PCa samples, tissue microarray*: PCa expression profile data from The Cancer Genome Atlas (TCGA) were obtained from the UCSC Xena database. Weighted Gene Co‐expression Network Analysis (WGCNA) was performed to identify gene modules significantly linked to PCa (*p* < 0.05). Subsequently, Kaplan‐Meier survival analysis and the log‐rank test were performed to assess the effect of candidate genes on disease‐free survival (DFS) and overall survival (OS) among patients diagnosed with PCa (P < 0.05). In parallel, expression datasets GSE62872 and GSE89223 were downloaded from the Gene Expression Omnibus (GEO) database. The genes identified by WGCNA were intersected with genes associated with DFS, genes associated with OS, and differentially expressed genes in GSE62872 and GSE89223 to screen for PCa‐related genes that are upregulated and associated with patient prognosis. All bioinformatics analyses were conducted using R software.

Due to a lack of commercially available normal prostate tissue wax blocks and fresh frozen paired specimens of PCa tissue and adjacent noncancerous tissue, we collected surgical samples from patients with pathologically confirmed PCa diagnosed in the Department of Urology, the First Affiliated Hospital of Anhui Medical University. The collected paired samples were fixed in formalin and embedded in paraffin (FFPE), and the proteins and RNA were extracted from the corresponding samples for subsequent experiments. All tissue samples were collected with written informed consent from patients and with approval from the Ethics Committee of the First Affiliated Hospital of Anhui Medical University (PJ2024‐06‐61). Clinical trial registration was not applicable because this study did not prospectively assign human participants to any health‐related intervention and did not report outcomes of an interventional human study.

The tissue microarray (TMA) used to validate CCT2 expression in PCa specimens was a paraffin‐embedded purchased from Aifang Biotechnology Co., Ltd. (Changsha, China) that contained 80 PCa tumor tissue samples. Subsequently, immunohistochemical staining was performed.

*RNA interference, plasmids, lentiviruses, and cell transfection*: siRNAs targeting human CCT2, EIF3F, and FOXA1 were synthesized from Tsingke (Beijing, China). Table S3 displays the complete primer sequences. The protein‐coding sequences of full‐length CCT2, Flag/Myc/His‐tagged CCT2, and Myc‐tagged truncation/mutant CCT2 were cloned and inserted into the vector pcDNA3.1‐PGK‐EGFP. The same vector was used to construct FASN, EIF3F, HA‐ubi, His‐ubi protein expression vectors (FASN, Myc‐FASN, EIF3F, Flag‐EIF3F, Myc‐EIF3F, Myc‐truncated EIF3F, Myc‐mutant EIF3F, HA/His‐ubi‐K6/11/27/29/33/48/63) and confirmed by Youbio (Chongqing, China). Moreover, the CCT2 and EIF3F coding sequences were subcloned and inserted into the vectors pET‐28a and pGEX‐6p‐1 with His or GST tags. The cells were seeded at 70%–90% confluency in 6‐well plates, transfected using Lipofectamine 3000 as specified by the manufacturer (L3000075, invitrogen), and incubated for 2–4 days at 37°C.

Validated sequences were cloned and inserted into the lentiviral vector pFU‐GW to produce short hairpin RNAs (shRNA‐CCT2) (GIDL5013115, genechem), and stably transfected cell lines were generated from PC‐3 cells transfected with pFU‐GW‐GFP‐CCT2. Lentiviral overexpression cells were generated by co‐transfecting the RM‐1 cell line with pLVX‐CMV‐luciferase‐CCT2 (GOSL5008864, genechem), followed by puromycin selection. Finally, qRT‐PCR and Western blotting was performed to assess the protein levels following knockdown or overexpression.

*Co‐immunoprecipitation (Co‐IP) assay and mass spectrometry analysis*: The cells were lysed directly in six‐well plates by adding 100 µL of RIPA lysis buffer to each well. To ensure sufficient protein yield, lysates from two identically transfected wells were pooled to obtain a 200 µL sample volume. The first step of the immunoprecipitation procedure was performed by incubating 200 µL of protein lysate with 3 µg of either control IgG (Invitrogen, 10400C) or a specific primary antibody for 12 h at 4°C with constant rotation. On the second day, the protein‐antibody complex was nurtured using 20 µL of protein A/G magnetic beads (Abbkine, BMR2080) at 4°C on a shaker for 20 min to facilitate binding. The complex was subsequently placed on a magnetic stand to pellet the beads. With the supernatant removed, the beads were resuspended in cell lysis buffer for Western blotting and IP (Beyotime, P0013). This process was repeated five times to minimize nonspecific binding. Finally, the immunoprecipitated proteins were eluted by resuspending the beads in 45 µL of 5× loading buffer, before denaturation for 5 min at 100°C. Following separation of the magnetic beads through a magnetic stand, the supernatant comprising the eluted proteins was collected for subsequent Western blotting.

Protein samples from both the IgG control and the antibody‐immunoprecipitated groups were separated by 10% SDS‐PAGE until the dye front had migrated approximately 1.5 cm into the separation gel. The target protein bands were excised and subjected to LC‐MS/MS analysis. Following in‐solution tryptic digestion, peptides were desalted using C18 StageTips (Thermo Scientific, USA) and analyzed with an Ultimate 3000 nanoLC system (Thermo Scientific, USA) coupled online to a Q Exactive Plus mass spectrometer (Thermo Scientific, USA).

*GST pull‐down assays*: For protein induction expression and purification, the constructed prokaryotic plasmids (GST vector, pGEX‐6p‐1‐GST‐EIF3F, pet‐28a‐His‐CCT2) were delivered to *Escherichia coli* BL21(DE3) (Solarbio, C1420) for shaking culture, and then isopropyl β‐D‐1‐thiogalactopyranoside (IPTG) (Beyotime, ST098) was supplemented to induce the expression of the recombinant protein. Total protein was extracted before and after induction. Recombinant GST‐tagged proteins were affinity‐purified using BeaverBeads GSH Magnetic Beads (Beaverbio, #70601), while His‐tagged proteins were isolated with BeaverBeads Ni‐NTA Magnetic Beads (Beaverbio, #70521P), according to the respective manufacturer protocols.

For pulldown experiments, the purified GST‐EIF3F and His‐CCT2 proteins were mixed and incubated for 12 h at 4°C. Next, protein A/G magnetic beads were introduced into the mixture, which was incubated at 4°C on a shaker for 3 h to capture the protein complexes. Finally, the protein complex was eluted from the beads for 5 min at 100°C. After that, the eluted proteins were resolved by SDS‐PAGE and analyzed by Western blotting.

SDS‐PAGE followed by silver staining (Biosharp, BL620A) was performed to evaluate the protein levels of samples collected at different stages (preinduction, postinduction, and post‐pulldown). The purified proteins were also analyzed with Coomassie Brilliant Blue staining solution (Beyotime, P0017) to assess purification efficiency.

*Ubiquitination assay*: For denaturing ubiquitination assays, cells were seeded in 6‐well plates and co‐transfected with the indicated plasmids, including Myc‐tagged FASN and His‐tagged ubiquitin. 48 h after transfection, cells were treated with the proteasome inhibitor MG132 for 6 h to prevent proteasomal degradation of ubiquitinated proteins. Cells were then washed with ice‐cold PBS and lysed in denaturing lysis buffer on ice for 30 min to disrupt non‐covalent protein–protein interactions.

Cell lysates were centrifuged to remove insoluble debris, and the supernatants were incubated with 50 µL Ni‐NTA magnetic beads (Beyotime, P2241) at 4°C with gentle rotation overnight. On the following day, the beads were collected using a magnetic stand, and non‐specifically bound proteins were removed by washing five times with denaturing wash buffer. Bound proteins were subsequently eluted using denaturing elution buffer, mixed with SDS loading buffer, and boiled for 10 min. Eluted samples were resolved by SDS‐PAGE and immunoblotted with anti‐Myc antibodies to detect polyubiquitinated target proteins.

*Turnover assay*: PC‐3 and DU145 cells were seeded into 6‐well plates and cultured to approximately 60%–70% confluence in complete medium supplemented with 10% fetal bovine serum (FBS). Cells were then treated with cycloheximide (CHX; 10 µg/mL; Selleck, S7418) to inhibit de novo protein synthesis [51]. Cells were harvested at the indicated time points (0, 4, 8, and 16 h) after CHX addition for immunoblot analysis. For the 0 h time point, cells were collected within 10–15 min after CHX addition.

At each time point, cells were washed three times with ice‐cold PBS and lysed on ice using RIPA buffer supplemented with a protease inhibitor cocktail. Equal amounts of total protein were subjected to SDS‐PAGE followed by Western blotting. Protein band intensities were quantified by densitometry using ImageJ software, normalized to β‐actin, and expressed relative to the 0 h time point. Decay constants (k) were obtained by linear regression of log_2_‐transformed normalized intensities. All experiments were performed with three independent biological replicates (n = 3), and statistical significance was assessed using a two‐tailed Student's *t*‐test.

Following treatment, the cells were harvested at specified time points (0, 4, 8, and 16 h) for Western blotting.

*Preparation of palmitate and orlistat*: palmitate (Selleck, S3794) was dissolved in DMSO to prepare a 10 mM stock solution. For palmitate rescue experiments, PC‐3 and DU145 cells were exposed to 200 µM palmitate for 48 h [52].

Orlistat (MCE, HY‐B0218) was dissolved in DMSO to generate a 10 mM stock solution. For in vivo treatment, orlistat was intraperitoneally administered to tumor‐bearing mice at 240 mg/kg until death, with the control groups receiving vehicle (DMSO) alone [53].

*Mouse models*: 4‐week‐old male C57BL/6 mice were offered by GemPharmatech Co., Ltd. (Nanjing, China) and housed under specific pathogen‐free (SPF) conditions for a 7‐day acclimation period, with unrestricted access to standard chow and autoclaved water. All animal experiments were reviewed and approved by the Institutional Animal Care and Use Committee of Anhui Medical University (Approval No. LLSC20242182) and carried out following the NIH Guide for the Care and Use of Laboratory Animals.

For the subcutaneous RM‐1 isograft model, 40 mice were randomly assigned into eight groups (n = 5 each). Subcutaneous isografts were established by injecting 100 µL of RM‐1 cell suspension (1 × 10^6^ cells in 1:1 PBS/Matrigel mixture; Absin, abs9492) into the dorsal flank. The tumors were measured every 7 days with digital calipers, and volumes were estimated using the formula: (length × width^2^) / 2. Drug administration was initiated once tumors became palpable. Orlistat was administered intraperitoneally at a dose of 240 mg/kg, and Y043‐8015 was administered intraperitoneally at 40 mg/kg, either alone or in combination, according to the indicated treatment schedules. Tumor growth was also recorded at the same timepoints using a small animal imaging system (Tanon, China) upon intraperitoneal administering 200 µL of D‐luciferin potassium salt solution (Beyotime, ST196), with images analyzed using Tanon System software. After all the mice were sacrificed, the tumors were excised, photographed under standardized conditions, and weighed. In addition, the tumors were further analyzed by H&E and immunohistochemistry.

For the bone metastasis model, mice were randomly assigned to eight groups (n = 5 per group). For the experimental bone metastasis model, RM‐1 cells (1 × 10^6^ cells in 20 µL PBS) were injected into the tibial marrow cavity of C57BL/6 mice via intratibial injection to establish bone metastatic lesions. Post‐injection, bioluminescence imaging was performed to monitor metastatic dissemination. After the mice were killed, the bone tissue morphology and related protein expression were evaluated by H&E and immunofluorescence.

For patient‐derived xenograft studies, freshly resected prostate cancer specimens were obtained and implanted subcutaneously into immunodeficient NSG mice to establish early‐passage PDX models. After tumor engraftment and expansion, PDX tumors with low or high CCT2 expression were selected and transplanted into recipient mice. Mice were then randomized into treatment groups receiving vehicle, orlistat, Y043‐8015, or combined therapy (n = 5 per group). Tumor growth was monitored by caliper measurement, and tumor volume and weight were recorded at the endpoint. Harvested PDX tumors were subjected to histological and immunohistochemical analyses.

*Statistical analyses*: Statistical analyses were conducted using R (v4.3.2), SPSS Statistics 25.0, and GraphPad Prism 9.0 software. Visual data representations were produced using ggplot2 (R) and the advanced charting functions of Prism. Raw data were examined for completeness and consistency before analysis, and quantitative values were normalized to the corresponding controls as indicated in the figure legends. Outliers were excluded only in cases of technical failure, which were explicitly stated. Data are presented as mean ± SD from at least three independent biological replicates. The exact sample size (n) is specified in the corresponding figure legends. Statistical tests were selected based on data distribution and experimental design. For comparisons between two groups, two‐tailed Student's *t*‐tests were used. For comparisons among multiple groups, one‐way ANOVA followed by appropriate post hoc tests was applied. Two‐way repeated‐measures ANOVA was used for tumor growth curves and time‐course experiments, as specified in the relevant figures. Paired two‐tailed *t*‐tests were used for paired experimental designs when applicable. Correlation analyses between protein expression levels were performed using Pearson's correlation coefficient (r) with 95% confidence intervals, assuming linear relationships. Survival analyses were conducted using the Kaplan‐Meier method, and differences between groups were evaluated using the log‐rank test. Categorical variables were compared using Pearson's χ^2^ test in SPSS. All analyses were conducted using two‐tailed tests, with statistical significance set at *p* < 0.05 (*p < 0.05, **p < 0.01, ***p < 0.001, ****p < 0.0001).

## Author Contributions

S.X., Y.Z., H.L., and S.Z. contributed equally to this work and are co‐first authors. C.L. conceived the project and designed the study. S.X., Y.Z., H.L., S.Z., and X.D. performed experiments, analyzed, and interpreted data. Z.Z., B.W., H.W., and L.Z. supervised experiments and interpreted data. S.X., Q.X., and M.F. collected clinical samples and analyzed data. S.X. wrote the original manuscript. L.Z. and C.L. revised and edited the manuscript. All authors discussed the results and commented on the manuscript.

## Conflicts of Interest

The authors declare no competing interests.

## Supporting information




**Supporting File**: advs75915‐sup‐0001‐SuppMat.docx.

## Data Availability

The data that support the findings of this study are available from the corresponding author upon reasonable request.
