# Supplementary material for: CCT2 Promotes Prostate Cancer Progression Through EIF3F‐Dependent Stabilization of FASN
Source: Adv Sci (Weinh). 2026 Jun 3;13(48):e75915. doi: 10.1002/advs.75915 (PMC13336740; doi:10.1002/advs.75915)
Supplement: Supplementary file 1 [file ADVS-13-e75915-s001.docx]

**CCT2 promotes prostate cancer progression through EIF3F-dependent stabilization of FASN**

*Shun Xu**^1,2,3#^, Yifan Zhang^1,2,3#^, Haolin Li^5#^, Shengyu Zhao**^1,2,3#^, Xiaoran Dai^4^, Qili Xu^1,2,3^, Mintian Fei**^1,2,3^, Chun Li^1,2,3^, Zhihui Zou^1,2,3^, Baojun Wang^1,2,3^, Li Zhang**^1,2,3^, Hui Wang^1,2,3*^, Ligang Zhang^1,2,3*^, Chaozhao Liang^1,2,3*^*

^1^Department of Urology, the First Affiliated Hospital of Anhui Medical University, Anhui Medical University, Hefei, Anhui, China.

^2^Institute of Urology, Anhui Medical University, Hefei, Anhui, China.

^3^Anhui Province Key Laboratory of Urological and Andrological Diseases Research and Medical Transformation, Anhui Medical University, Hefei, Anhui, China.

^4^Department of Clinical Laboratory, The First Affiliated Hospital of Anhui Medical University, Hefei, China.

^5^Department of Urology, The First Affiliated Hospital of Kunming Medical University, Kunming, Yunnan, China.

^*^Correspondence:

Chaozhao Liang

Department of Urology, the First Affiliated Hospital of Anhui Medical University, No. 218 Jixi Road, Hefei 230022, Anhui, China

Email: liang_chaozhao@ahmu.edu.cn

Ligang Zhang

Department of Urology, the First Affiliated Hospital of Anhui Medical University, No. 218 Jixi Road, Hefei 230022, Anhui, China

Email: Lgzhang08@163.com

Hui Wang

Department of Urology, the First Affiliated Hospital of Anhui Medical University, No. 218 Jixi Road, Hefei 230022, Anhui, China

Email: whayd@sina.cn

^#^Shun Xu, Yifan Zhang, Haolin Li and Shengyu Zhao contributed equally to this work.

**Supporting Information**

**Supplementary Methods**

*Cell culture：*All the cell lines were obtained from Wuhan Pricella Biotechnology Co., Ltd (Wuhan, China) in September 2023, authenticated by STR analysis, routinely tested for Mycoplasma contamination with negative results, and maintained at 37℃ and 5% CO_2_. The use of these cell lines is essential for studying the role of CCT2 in prostate cancer proliferation and metastasis, and does not affect the validity of the conclusions. PCa cell lines (PC-3, DU 145, C4-2, LNCaP, and RM-1) were cultured in RPMI 1640 medium. Human normal prostate epithelial cell (RWPE-1) was cultured in Keratinocyte Medium (KM). HEK-293T cell line was cultured in Dulbecco’s modified Eagle’s medium (DMEM). All the media (RPMI 1640 and DMEM) were supplemented with 10% fetal bovine serum (FBS) and 1**×**Penicillin-Streptomycin solution. The RRID identifiers of the cell lines used in this study are as follows: PC-3 (RRID: CVCL_0035), DU145 (RRID: CVCL_0105), C4-2 (RRID: CVCL_4782), LNCaP (RRID: CVCL_0395), RM-1 (RRID: CVCL_B459), RWPE-1 (RRID: CVCL_3791), and HEK-293T (RRID: CVCL_0063).

*Integrated transcriptomic and lipidomic profiling: combining RNA-seq with* *broad-spectrum metabolomic analysis**：* Total RNA was extracted using TRIzol reagent. RNA quality and integrity were assessed by NanoDrop spectrophotometer (Thermo Fisher Scientific, USA) and Agilent 2100 Bioanalyzer (Agilent Technologies, USA). Poly(A)+ mRNA was isolated through oligo(dT)-conjugated magnetic beads to purify mRNA. Ribosomal RNA was subsequently removed by the RIbo-Zero kit according to the manufacturer's protocol to enrich mRNA (Illumina, USA). After passing quality control assessments, the Small RNA Library Preparation Kit was used to construct the library (Illumina, USA). SOAPnuke (V2.1.0) was performed to filter the raw sequencing data. High-quality clean reads were then aligned to the reference genome using HISAT2 (v2.1.0), followed by transcriptome alignment with Bowtie2 (v2.3.5) against reference transcript sequences. Transcript abundance was quantified using RSEM (v1.3.3) with bowtie2 alignment results as input. Gene expression levels were normalized and expressed as FPKM (Fragments Per Kilobase of transcript per Million mapped reads). Statistical analysis of differentially expressed genes was performed using DESeq2 (v1.22.2) and edgeR (v3.6.8) packages in R. Genes with adjusted *p < 0.05* and absolute log2 fold change (|log2FC|) > 1 were considered statistically significant. Functional enrichment analysis was performed using Gene Ontology (GO) and Kyoto Encyclopedia of Genes and Genomes (KEGG) databases. Significantly enriched terms were identified with a threshold of *p < 0.05* after multiple testing correction.

Global lipid profiling was performed using liquid chromatography coupled with tandem mass spectrometry (LC-MS/MS) to quantitatively compare lipidomic profiles between PC-3 cells transduced with control lentivirus (Lv-Ctrl) versus CCT2-knockdown cells (Lv-sh-CCT2). Briefly, to each sample, 1 mL of ice-cold extraction solvent spiked with internal standard mixture was added. The mixture was vortexed vigorously for 15 min at 4°C, followed by addition of ice-cold ultrapure water. After vortex mixing (1 min) and centrifugation (12,000 × g, 10 min, 4°C), the upper organic phase was carefully transferred to a clean tube. The extract was evaporated to dryness in CentriVap Concentrator (Labconco, USA), and the residue was reconstituted in mobile phase (acetonitrile: isopropanol = 1:1). The sample extracts were analyzed using an LC-ESI-MS/MS system. Differential metabolites were determined by VIP (VIP > 1) and P-value (*P < 0.05*). VIP values were extracted from OPLS-DA result, and generated using R package MetaboAnalystR. The data was log transform (log_2_). Metabolite identification was performed by matching experimental MS/MS spectra against the KEGG Compound database. Statistically significant metabolites were subsequently mapped to the KEGG Pathway database. Pathway enrichment analysis was conducted through Metabolite Set Enrichment Analysis (MSEA), with statistical significance assessed via hypergeometric test (*p<0.05*).

*Western blotting (WB) ：* Total proteins were extracted from cells or tissues using RIPA Lysis Buffer (Beyotime, P0013B) supplemented with 1 mM PMSF (Beyotime, ST505) protease inhibitor. Protein concentrations were quantified using a BCA assay kit (Beyotime, P0012S). Equal amounts of protein were separated by SDS-PAGE and subsequently transferred to PVDF membranes (0.45μm) via wet electroblotting. Following transfer, PVDF membranes were blocked with 5% bovine serum albumin (Beyotime, ST023) for 1 h at room temperature. Membranes were then incubated with primary antibodies at 4°C overnight. After extensive washing, membranes were probed with horseradish peroxidase (HRP)-conjugated secondary antibodies (Elabscience, E-AB-1003, 1:5000) for 1 h at room temperature with shaking. Protein bands were visualized using BeyoECL Moon chemiluminescent substrate (Beyotime, P0018FS) and imaged using the Tanon-4100 (Tanon, China) imaging system. Band intensity quantification was performed using ImageJ software. The specific primary antibodies including their sources, catalog numbers, and dilution ratios, are detailed in Table S2, Supporting Information.

*RNA extraction,* *reverse transcription (RT), and quantitative reverse transcriptase (qRT‒PCR)**：* Total RNA was extracted from cells or tissues using TRIzol reagent (Invitrogen, #15596026) according to the manufacturer protocol. RNA purity and concentration were determined with a NanoDrop microvolume spectrophotometer (Thermo Fisher Scientific, USA). Complementary DNA (cDNA) was synthesized using ToloScript All-in-one RT EasyMix (Tolobio, #22107). Quantitative PCR amplification was subsequently performed using 2**×** Q3 SYBR qPCR Master Mix (Tolobio, #22204) on an ABI 7500 PCR machine (Applied Biosystems, USA) with gene-specific primers. Fluorescence data were collected and analyzed using the 2^-ΔΔCt^ method for relative quantification. All samples were analyzed in triplicate. Gene expression levels were normalized to those of GAPDH or β-actin. All primers were designed and synthesized by Sangon Biotech (Shanghai, China). The complete primer sequences are provided in Table S4, Supporting Information.

*Proximity ligation assay (PLA)：*PC-3 cells were seeded onto sterile glass coverslips placed in 6-well plates. After cell attachment and reaching an appropriate confluence, cells were treated as indicated. Cells were then fixed with 4% paraformaldehyde at room temperature for 10 min. Following PBS washes, cells were incubated with the blocking solution provided in the PLA kit (sigma, DUO92002; DUO92004; DUO92014) at 37 °C for 30 min to minimize nonspecific background signals. After blocking, cells were incubated overnight at 4 °C with primary antibodies raised in different species. On the following day, cells were washed with PBS and incubated with species-specific PLA probes (PLUS and MINUS) at 37 °C for approximately 1 h. Ligation and rolling-circle amplification reactions were subsequently performed according to the manufacturer’s instructions. Cell nuclei were counterstained with DAPI. Images were acquired using a confocal fluorescence microscope under identical imaging settings, and PLA signals were used to assess the intracellular proximity between proteins of interest.

*Nascent protein synthesis assay:* Cells were treated as indicated, after which the culture medium was removed and cells were gently washed once with PBS. Pre-warmed 1× HPG working solution (2 mL per well) was then added, and cells were incubated at 37 °C for 0.5 h to allow incorporation of HPG into newly synthesized proteins. Following incubation, the HPG solution was removed and cells were fixed at room temperature for 15 min. After fixation, cells were washed three times with wash buffer (3–5 min each) and subsequently permeabilized at room temperature for 10–15 min. Cells were then washed again 1–2 times with wash buffer. Click reaction solution (0.5 mL per well) was added and incubated for 30 min at room temperature in the dark to fluorescently label HPG-incorporated proteins. After completion of the Click reaction, cells were washed three times with wash buffer. Cell nuclei were counterstained with 1× Hoechstvx solution (1 mL per well) for 10 min at room temperature in the dark, followed by three additional washes. Fluorescence images were acquired using a fluorescence microscope under identical imaging settings to evaluate nascent protein synthesis.

*Preparation of Small-Molecule Compounds**：*The structure of CCT2-EIF3F complex was obtained from alphafold3 and analysed the interaction sites of CCT2 for use as the receptor structure for molecular docking studies. The substrates were constructed and hydrogenated, and then underwent structural optimization using the molecular orbital package program. Molecular docking was conducted using the Autodock Vina 1.2.0 software, with the docking box encompassing the active site and a docking time of 3 min. All other parameters were maintained at their default settings. Finally, nine small-molecule compounds that met the selection criteria (5137-3728, Y043-8015, C370-4100, Y512-1716, 8002-6276, E683-0240, 8015-8093, 8002-6305, and 8003-5590) were obtained from ChemDiv (USA). To evaluate their cytotoxicity, PC-3 and DU145 prostate cancer cell lines were treated with each compound at increasing concentrations (0, 10, 20, 30, 40, and 50 μM), and the half-maximal inhibitory concentration (IC50) values were calculated. The compound with the highest inhibitory efficiency was identified for further analysis. Subsequently, PC-3, DU145, and 293T cells were treated with the selected compound at concentration gradients of 0, 1/4 IC50, 1/2 IC50, and IC50 to assess its dose-dependent effects.

*DNA affinity pull-down assay and mass spectrometry analysis**：* The PC-3 cell line was utilized for DNA pull-down assays following the DNA pull-down kit's protocol (Baiwei, BW2504). Biotinylated DNA probes were conjugated to streptavidin-coated magnetic beads in binding buffer for 30 min at room temperature with rotation. Nuclear protein extracts were treated with DNase to eliminate contaminating nucleic acids, followed by pre-clearing with bare magnetic beads. DNA-protein pull-down was performed by incubating the pre-cleared nuclear lysates with probe-conjugated beads overnight at 4°C with gentle rotation. Protein samples were analyzed by silver staining and subsequently subjected to liquid chromatography-mass spectrometry (LC-MS) for protein identification.

*Cell proliferation and colony formation：* For the cell viability assay, PC-3 and DU145 cells were plated at a density of 3,000-5,000 cells per well in 96-well plates. To minimize edge effects due to evaporation, PBS was added to the outer wells. Cell viability was evaluated using a Cell Counting Kit-8 (CCK-8) (GlpBio, GK10001) at 0, 1, 2, 3, and 4 days. The detection reagent was prepared by diluting the CCK-8 solution with serum-free medium at a 1:10 ratio. After incubation at 37°C for 1 hour, the optical density (OD) was measured at 450 nm and 650 nm using a microplate reader.

For the colony formation assay, cells were seeded at a density of 500 cells per well in 6-well plates and maintained in a humidified incubator (37°C, 5% CO₂) for 7-14 days. The assay was terminated when macroscopic colonies (≥50 cells per colony) were observed. Following culture termination, cells were washed with PBS, fixed with 4% paraformaldehyde (PFA) (Beyotime, P0099) for 15 min at room temperature, and stained with Crystal Violet Staining Solution (Beyotime, C0121) for 10 min. After thorough rinsing with water, plates were air-dried for observation. The number of clones was quantified using ImageJ software.

*Wound healing, and cell migration, invasion**：* In the Wound healing assay, confluent monolayers of PC-3, DU145 and RM-1 cells (approximately 80% density) in 6-well plates were scratched using a sterile 200 μL pipette tip held perpendicular to the plate surface. Then, the dislodged cells were washed with PBS, and the wound healing was monitored using an inverted phase-contrast microscope at 0h and 2h post-scratching. The wound width was quantified using ImageJ software by measuring the distance between wound edges.

Cell migration and invasion were assessed using Transwell chambers (Corning, #3464). For invasion assays, membranes were pre-coated with Matrigel for 4h (Absin, abs9492). Cells (5×10³/well) in serum-free medium were seeded in the upper chamber, filled with 200 μL of serum-free medium, and 800 μL of RPMI 1640 medium containing 10% FBS was added to the lower chamber. After 24 h or 48 h, migrated or invaded cells were fixed with 4% PFA for 15 min at room temperature and stained with Crystal Violet Staining Solution for 30 min, and non-migrated cells on the upper membrane surface were removed using pre-wet cotton swabs. Membranes were photographed under an inverted microscope and quantified using ImageJ software.

*Oil red O staining and* *measurement of lipid droplets:* In the Oil Red O experiment, PC-3 and DU145 cells were seeded in 6-well plates and cultured until reaching approximately 50% confluency, followed by lipid droplet staining using the Oil Red O stain kit (Solarbio, G1262). The Oil Red O staining solution was freshly prepared and filtered through three layers of qualitative filter paper to remove particulate impurities. Cells were processed as follows: (1) culture medium was removed and washed thrice with PBS; (2) fixed with 4% PFA for 30 min at room temperature; (3) washed with distilled water (2-3 washes); (4) treated with 60% isopropanol for 30 sec to enhance lipid droplet staining; (5) incubated with freshly prepared Oil Red O solution for 20 min at room temperature; and (6) counterstained with Mayer’s hematoxylin for 1-2 min for nuclear visualization. Finally, Stained cells were observed and captured under a microscope.

The intracellular lipid droplet content in PC-3 and DU145 cells was assessed using the lipophilic fluorescent dye BODIPY 493/503 (Invitrogen, D2191), prepared as a 2 μM working solution.

For lipid droplet visualization, cells were washed twice with PBS and fixed with 4% PFA for 20 min at room temperature. Following fixation, cells were stained with 2 μM BODIPY 493/503 in PBS for 15 min at room temperature protected from light. After three PBS washes, nuclei were counterstained with 10 μg/mL Hoechst 33342 (MCE, HY-15559) for 5 min in the dark. Images were acquired using a Leica confocal microscope.

For quantitative lipid droplet assessment, Briefly, cells were washed twice with PBS and stained with 2 μM BODIPY 493/503 for 15 min at room temperature in the dark. After trypsinization and centrifugation, cells were resuspended in PBS. Flow cytometry analysis was immediately conducted using a Beckman flow cytometer (Beckman Coulter, USA). Data were processed using FlowJo (v10.8.1).

*Immunohistochemical (IHC), and immunofluorescence (IF) staining:*

Immunohistochemistry: Antigen retrieval was performed in 1 mM EDTA for 20 min at 95°C, and nonspecific binding was blocked with 5% BSA in PBS for 30 min at 37°C. Sections were then incubated with primary antibodies at 4°C overnight. Following PBS washes, HRP-conjugated secondary antibodies were applied for 1 h at room temperature. Immunostaining was developed using 3,3'-diaminobenzidine (DAB) (Beyotime, P0202) for precisely 10 minutes at room temperature, followed by nuclear counterstaining with Mayer's hematoxylin (Beyotime, C0107). High-resolution whole-slide imaging was performed using the digital slice scanner (KFBIO, China).

Immunofluorescence: Sterile cell culture slides (Biosharp, BS-25-RC) were placed in 6-well plates and seeded with cells at appropriate density. Following 24-hour incubation to ensure cellular adherence, cells were fixed with 4% PFA in PBS for 20 min at room temperature. Permeabilization was subsequently performed using 0.5% Triton X-100 (Beyotime, P0096) in PBS for 10 min at room temperature. Following blocking with 5% BSA in PBS for 1 h at room temperature, slides were incubated with primary antibodies at 4°C overnight. Alexa Fluor-conjugated secondary antibodies were applied and incubated for 1 h at room temperature under light-protected conditions. Slides were mounted with Antifade Mounting Medium with DAPI (Beyotime, P0131). The sections were photographed using a Leica confocal microscope.

*Chromatin immunoprecipitation (ChIP) and dual-luciferase reporter assays:* The chromatin immunoprecipitation (ChIP) assay was performed in the PC-3 cell line according to the instructions of the chromatin immunoprecipitation kit (Baiwei, BW2501). Briefly, the cells were crosslinked with 1% formaldehyde. Chromatin was isolated and sheared into 200-500 bp fragments using a sonicator (Diagenode, Belgium). Immunoprecipitation was performed overnight at 4°C using anti-FOXA1 or IgG antibodies, with subsequent protein G magnetic bead capture. Immunoprecipitated DNA-protein complexes were eluted in elution buffer, followed by reversal of cross-linking. Then, the DNA was purified and precipitated. qRT‒PCR was performed, and the resulting PCR products were visualized by agarose gel electrophoresis. The complete primer sequences are provided in Table S5, Supporting Information.

For the dual-luciferase reporter assays, HEK-293T cells were seeded in 24-well plates and cultured until they reached 70–80% confluency. Subsequently, the cells were co-transfected with plasmids bearing the wild-type or mutant pGL3-CCT2 promoter, a Renilla luciferase reporter plasmid (pRL-TK), and pcDNA3.1-FOXA1 plasmids. At 48 h post-transfection, luciferase activity was quantified using the Dual Luciferase Reporter Gene Assay Kit (YEASEN, #11402ES60) on a multifunctional microplate reader (BioTek, USA). The relative promoter activity was calculated as the ratio of Fluc to Rluc luminescence values (RLU), normalized to empty vector control transfections.

*Microscale thermophoresis (MST)**:* His-CCT2 protein expression was successfully induced with 0.3 mM IPTG at 16 °C, followed by purification and quantification using the Bradford protein assay (Bradford Protein Assay Kit，P0006). After quality assessment, the purified protein was deemed suitable for subsequent MST analysis. Fluorescent labeling was performed using the Monolith™ RED-NHS second-generation protein labeling kit (NanoTemper, MO-L002), and the labeled protein was immediately placed on ice for MST experiments. Binding interactions between His-CCT2 and Y043-8015 were assessed by MST, and the resulting data were analyzed using MO.Affinity Analysis software.

*Surface plasmon resonance (SPR)*: Assays were performed on a Biacore 8K system (Cytiva) equipped with a CM5 sensor chip. The chip surface was activated by injecting a freshly prepared 1:1 mixture of 400 mM N-ethyl-N′-(3-dimethylaminopropyl)carbodiimide (EDC) and 100 mM N-hydroxysuccinimide (NHS) at a flow rate of 10 μL min⁻¹ for 420 s. CCT2 was then diluted to 20 μg mL⁻¹ in immobilization buffer and immobilized onto the sample flow cell (Fc2) at 10 μL min⁻¹, reaching an immobilization level of approximately 12 600 RU, whereas the reference flow cell (Fc1) was left blank. Residual activated esters were blocked by injection of 1 M ethanolamine hydrochloride (pH 8.5) at 10 μL min⁻¹ for 420 s. For kinetic analysis, 20 μM EIF3F protein was premixed with Y043-8015 at seven different concentrations (0.31–20 μM) in the same running buffer and incubated at 4 °C for 30 min. The mixtures were then sequentially injected over Fc1 and Fc2 at a flow rate of 20 μL min⁻¹, with an association phase of 100 s and a dissociation phase of 180 s. Seven binding cycles were performed in ascending order of compound concentration, and the sensor surface was regenerated after each cycle to restore the baseline. The resulting sensorgrams were analyzed using Biacore Insight Evaluation Software (Cytiva) and fitted with a 1:1 Langmuir binding model.

**Supplementary Figures**

**Supplementary Figure 1. WGCNA and single-cell analysis reveal PCa-related gene modules and cell-type compositions.**

(A) WGCNA of TCGA prostate cancer dataset. Left: scale-free topology model fit and mean connectivity plots indicate that a soft threshold power of 6 was selected. Middle: hierarchical clustering dendrogram of genes, with modules represented by distinct colors. Right: module-trait relationship heatmap reveals significant correlation between the module (MEblue, MEyellow, and MEblack) and prostate cancer progression-related traits. (B) Dot plot displays canonical marker gene expression across major cell types. Dot size indicates the proportion of expressing cells; color reflects average expression. (C) Quantitative densitometric analysis of CCT2 protein expression in normal prostate epithelial cells and prostate cancer cell lines. Protein levels were normalized to GAPDH. Data are presented as mean ± SD from three independent biological replicates (n = 3). Statistical significance was assessed using one-way ANOVA. (D) Paired quantitative densitometric analysis of CCT2 protein expression in matched normal prostate tissues and primary prostate cancer tissues (n = 12 pairs). Statistical significance was assessed using a paired two-tailed Student’s *t*-test. For quantitative panels with significance annotations, ns indicates *P* > 0.05, **P* < 0.05, ****P* < 0.001.

**Supplementary Figure 2.** **CCT2 overexpression promotes prostate cancer cell proliferation, migration, and lipid accumulation.**

(A, B) Western blotting and qRT-PCR confirm successful overexpression of CCT2 in PC-3 and DU145 cells. Data are presented as mean ± SD from three independent biological replicates (n = 3). Statistical significance was assessed using two-tailed unpaired Student’s *t*-tests. (C, D) CCK-8 assays were performed to evaluate cell viability following CCT2 overexpression. Data are presented as mean ± SD from three independent biological replicates (n = 3). Statistical significance was assessed using two-way ANOVA. (E, H) Wound healing assays were used to assess the migratory behavior of CCT2-overexpressing cells after 24 hours. Data are presented as mean ± SD from three independent biological replicates (n = 3). Statistical significance was assessed using two-tailed unpaired Student’s t-tests. (F, G) Colony formation assays were conducted to measure the clonogenic potential of PCa cells overexpressing CCT2. Data are presented as mean ± SD from three independent biological replicates (n = 3). Statistical significance was assessed using two-tailed unpaired Student’s t-tests. (I–K) Transwell migration and Matrigel invasion assays were performed to analyze cell migration and invasion capabilities, respectively. Data are presented as mean ± SD from three independent biological replicates (n = 3). Statistical significance was assessed using two-tailed unpaired Student’s t-tests. (L) Validation of CCT2 knockdown efficiency and its effect on PC-3 cell proliferation. Data are presented as mean ± SD from three independent biological replicates (n = 3). Statistical significance was assessed using two-tailed unpaired Student’s t-tests for knockdown validation and two-way ANOVA for growth curves. (M) Volcano plot displaying differentially expressed lipid species between Lv-sh-CCT2 and control cells. (N) GO enrichment of DEGs indicates significant upregulation of metabolic process-related pathways. (O) Heatmap shows global changes in lipid species abundance, grouped by lipid classes, between Lv-CCT2 and control cells. (P) Flow cytometry analysis of FITC-BODIPY-stained PC-3 and DU145 cells showing relative lipid droplet content. Data are presented as mean ± SD from three independent biological replicates (n = 3). Statistical significance was assessed using one-way ANOVA. (Q) Oil Red O and BODIPY 493/503 staining were used to visualize intracellular lipid droplets in CCT2-overexpressing and control cells. Data are presented as mean ± SD from three independent biological replicates (n = 3). Statistical significance was assessed using two-tailed unpaired Student’s *t*-tests. For quantitative panels with significance annotations, **P* < 0.05, ****P* < 0.001.

**Supplementary Figure 3. CCT2 interacts with and regulates FASN expression in prostate cancer cells.**

(A) Representative confocal immunofluorescence images showing the subcellular localization and colocalization of Flag-tagged CCT2 (red) and Myc-tagged FASN (green) in PC-3 and DU145 cells. Scale bars, 10 μm. (B) Relative protein expression levels of CCT2 and FASN in PC-3 and DU145 cells following transfection with si-Ctrl, or si-CCT2#1 and si-CCT2#3. Data are presented as mean ± SD from three independent biological replicates (n = 3). Statistical significance was assessed using one-way ANOVA. (C) Relative protein expression levels of CCT2 and FASN in PC-3 and DU145 cells transduced with plvx-vector or plvx-CCT2. Data are presented as mean ± SD from three independent biological replicates (n = 3). Statistical significance was assessed using two-tailed unpaired Student’s *t*-tests. (D) Relative protein expression levels of CCT2 and FASN in cells treated with si-Ctrl or si-CCT2 in the presence or absence of the proteasome inhibitor MG132. Data are presented as mean ± SD from three independent biological replicates (n = 3). Statistical significance was assessed using one-way ANOVA. For quantitative panels with significance annotations, **P* < 0.05, ***P* < 0.01.

**Supplementary Figure 4. EIF3F stabilizes FASN protein by suppressing proteasomal degradation without affecting mRNA expression.**

(A) The results of the three algorithms (CytoHubba, MCODE, and CytoNCA). (B) Silver staining and LC-MS/MS confirmed the interaction among FASN, CCT2, and EIF3F, with EIF3F identified by peptide spectrum matches. (C) Representative confocal immunofluorescence images showing the subcellular localization and colocalization of Flag-tagged EIF3F (red) and Myc-tagged FASN (green) in PC-3 and DU145 cells. Scale bars, 10 μm. (D) Relative protein expression levels of EIF3F and FASN in PC-3 and DU145 cells following transfection with EIF3F knockdown or EIF3F overexpression. Data are presented as mean ± SD from three independent biological replicates (n = 3). Statistical significance was assessed using one-way ANOVA for si-Ctrl/si-EIF3F comparisons and two-tailed unpaired Student’s t-tests for plvx-vector/plvx-EIF3F comparisons. (E) qRT-PCR analysis of FASN mRNA levels in PC-3 cells after modulation of EIF3F expression. Data are presented as mean ± SD from three independent biological replicates (n = 3). Statistical significance was assessed using one-way ANOVA for si-Ctrl/si-EIF3F comparisons and two-tailed unpaired Student’s t-tests for plvx-vector/plvx-EIF3F comparisons. (F) Representative images of nascent protein synthesis in PC-3 cells measured by HPG (L-homopropargylglycine) incorporation followed by Click-iT labeling with Alexa Fluor 488. Scale bars, 20 μm. (G) Quantification of relative fluorescence intensity from HPG incorporation assays. Data are presented as mean ± SD from three independent biological replicates (n = 3). Statistical significance was assessed using one-way ANOVA. (H) CHX chase assays were performed to evaluate changes in FASN protein stability following EIF3F knockdown or overexpression. Protein band intensities were quantified by densitometry, normalized to β-actin, and expressed relative to the 0 h time point. Decay constants (k) were obtained by linear regression of log₂-transformed normalized intensities. Data are presented as mean ± SD from three independent biological replicates (n = 3). Statistical significance was determined using a two-tailed Student’s *t*-test. (I) Western blotting analysis evaluating the effect of the proteasome inhibitor MG132 on FASN protein levels in the presence or absence of EIF3F. Data are presented as mean ± SD from three independent biological replicates (n = 3). Statistical significance was assessed using one-way ANOVA. For quantitative panels with significance annotations, ns indicates *P* > 0.05, **P* < 0.05, ***P* < 0.01,****P* < 0.001, *****P* < 0.0001.

**Supplementary Figure5. CCT2 and EIF3F do not regulate each other’s expression or protein stability.**

(A) Western blotting analysis of EIF3F and CCT2 expression in PC-3 and DU145 cells transfected with si-Ctrl, si-CCT2#1 or si-CCT2#3. Quantification of relative protein levels is shown on the right. Data are presented as mean ± SD from three independent biological replicates (n = 3). Statistical significance was assessed using one-way ANOVA. (B) Western blotting analysis of EIF3F and CCT2 expression in PC-3 and DU145 cells transduced with plvx-vector or plvx-CCT2. Quantification of relative protein expression is shown on the right. Data are presented as mean ± SD from three independent biological replicates (n = 3). Statistical significance was assessed using two-tailed unpaired Student’s t-tests. (C, D) CHX chase assays assessing EIF3F protein stability in PC-3 cells following CCT2 knockdown or CCT2 overexpression. Representative immunoblots and corresponding protein decay curves are shown. Data are presented as mean ± SD from three independent biological replicates (n = 3). Protein half-life was quantified by linear regression of log-transformed protein levels over time, and decay rates were compared between groups using two-tailed Student’s *t*-tests. (E, F) CHX chase assays assessing EIF3F protein stability in DU145 cells following CCT2 knockdown or CCT2 overexpression. Representative immunoblots and corresponding protein decay curves are shown. Data are presented as mean ± SD from three independent biological replicates (n = 3). Protein half-life was quantified by linear regression of log-transformed protein levels over time, and decay rates were compared between groups using two-tailed Student’s *t*-tests. (G) Western blotting analysis of EIF3F and CCT2 expression in PC-3 and DU145 cells transfected with si-Ctrl, si-EIF3F#2 or si-EIF3F#3. Quantification of relative protein levels is shown on the right. Data are presented as mean ± SD from three independent biological replicates (n = 3). Statistical significance was assessed using one-way ANOVA. (H) Western blotting analysis of EIF3F and CCT2 expression in PC-3 and DU145 cells transduced with plvx-vector or plvx-EIF3F. Quantification of relative protein expression is shown on the right. Data are presented as mean ± SD from three independent biological replicates (n = 3). Statistical significance was assessed using two-tailed unpaired Student’s *t*-tests. (I, J) CHX chase assays assessing CCT2 protein stability in PC-3 cells following EIF3F knockdown or EIF3F overexpression. Representative immunoblots and corresponding protein decay curves are shown. Data are presented as mean ± SD from three independent biological replicates (n = 3). Protein half-life was quantified by linear regression of log-transformed protein levels over time, and decay rates were compared between groups using two-tailed Student’s *t*-tests. (K, L) CHX chase assays assessing CCT2 protein stability in DU145 cells following EIF3F knockdown or EIF3F overexpression. Representative immunoblots and corresponding protein decay curves are shown. Data are presented as mean ± SD from three independent biological replicates (n = 3). Protein half-life was quantified by linear regression of log-transformed protein levels over time, and decay rates were compared between groups using two-tailed Student’s *t*-tests. For quantitative panels with significance annotations, ns indicates *P* > 0.05, **P* < 0.05, ***P* < 0.01.

**Supplementary Figure 6.** **CCT2 physically interacts with EIF3F and enhances FASN ubiquitination through EIF3F-dependent mechanisms.**

(A) Co-IP assays confirmed the physical interaction between Flag-CCT2 and His-EIF3F in HEK-293T cells and PCa cell lines (PC-3 and DU145). (B) Representative confocal immunofluorescence images showing the subcellular localization and colocalization of Flag-tagged CCT2 (red) and His-tagged EIF3F (green) in PC-3 and DU145 cells. Scale bars, 10 μm. (C) Proximity ligation assay (PLA) detecting endogenous CCT2–EIF3F interactions in PC-3 cells. Scale bars, 10 μm. (D) SDS-PAGE analysis of purified recombinant proteins. GST-EIF3F and His-CCT2 were successfully expressed and purified from E. coli as shown by Coomassie blue staining. (E) Summary table of seven predicted interacting regions between CCT2 and EIF3F identified via molecular docking and structural modeling, highlighting conserved residues potentially mediating the interaction. (F) Structural visualization of the seven candidate binding regions. Amino acid residues within predicted interaction interfaces are labeled and color-coded: CCT2 residues in green, EIF3F residues in cyan. Insets display atomic-level contacts in each region. Among them, Region 1 (CCT2: 298, 494, 498, 502; EIF3F: 207, 208, 210) showed the strongest binding potential based on modeling. (G) Quantification of Myc-FASN protein levels in cells expressing increasing amounts of Flag-CCT2 (low, medium, and high). Data are presented as mean ± SD from three independent biological replicates (n = 3). Statistical significance was assessed using one-way ANOVA. (H) Ni–NTA pull-down assays detecting ubiquitinated FASN in cells transfected with His-tagged ubiquitin (K48), Flag-CCT2, and the indicated siRNAs targeting EIF3F, USP2, or TRIM21.For quantitative panels with significance annotations, ns indicates *P* > 0.05, **P* < 0.05, ***P* < 0.01.

**Supplementary Figure 7.** **Cytotoxicity screening of candidate small-molecule compounds in PCa cell lines.**

(A, B) CCK-8 assays assessing the cytotoxicity of nine candidate small-molecule compounds in PC-3 (A) and DU145 (B) cells. Data are presented as mean ± SD from three independent biological replicates (n = 3). Dose–response curves were fitted by nonlinear regression, and IC50 values were calculated accordingly. Y043-8015 exhibited the lowest IC50 in both cell lines, indicating potent anti-proliferative activity. (C) SDS-PAGE analysis showing purification of His-tagged CCT2 protein expressed in E. coli.

**Supplementary Figure 8. FOXA1 positively correlates with and transcriptionally regulates CCT2 expression in prostate cancer.**

(A) Correlation analysis of FOXA1 and CCT2 gene expression in prostate cancer samples. (B) IHC images show elevated nuclear FOXA1 and cytoplasmic CCT2 in tumor tissues compared to adjacent normal tissues, with a positive correlation between them. (C) Quantitative analysis of FOXA1 and CCT2 relative protein expression in PC-3 and DU145 cells following transfection with si-Ctrl, si-FOXA1#2, and si-FOXA1#3. Data are presented as mean ± SD from three independent biological replicates (n = 3). Statistical significance was assessed using one-way ANOVA. (D) qRT-PCR analysis of CCT2 relative mRNA expression in PC-3 and DU145 cells following FOXA1 knockdown. Data are presented as mean ± SD from three independent biological replicates (n = 3). Statistical significance was assessed using one-way ANOVA. (E) qRT–PCR analysis of EIF3F and FASN relative mRNA levels in PC-3 and DU145 cells following FOXA1 knockdown. Data are presented as mean ± SD from three independent biological replicates (n = 3). Statistical significance was assessed using one-way ANOVA. (F) Western blotting analysis of FOXA1 and EIF3F relative protein levels in PC-3 and DU145 cells transfected with si-Ctrl or si-FOXA1. Data are presented as mean ± SD from three independent biological replicates (n = 3). Statistical significance was assessed using one-way ANOVA. (G) Western blotting analysis of FOXA1 and FASN relative protein levels in PC-3 and DU145 cells following FOXA1 knockdown. Data are presented as mean ± SD from three independent biological replicates (n = 3). Statistical significance was assessed using one-way ANOVA. For quantitative panels with significance annotations, ns indicates *P* > 0.05, **P* < 0.05, ***P* < 0.01,****P* < 0.001, *****P* < 0.0001.

**Supplementary Figure 9. Pharmacological inhibition with Orlistat and Y043-8015 attenuates CCT2-associated aggressive phenotypes in prostate cancer cells.**

(A) Representative images and quantification of colony formation assays in PC-3 and DU145 cells expressing Lv-Ctrl or Lv-OE-CCT2. Cells were treated with vehicle, Orlistat, Y043-8015, or the combination as indicated. (B) Transwell migration and invasion assays of PC-3 and DU145 cells with Lv-Ctrl or Lv-OE-CCT2 under the indicated treatments. (C) Wound-healing assays assessing cell migratory capacity in PC-3 and DU145 cells expressing Lv-Ctrl or Lv-OE-CCT2 following treatment with vehicle, Orlistat, Y043-8015, or the combination. Representative images at 0 h and 24 h are shown. (D) Representative Oil Red O staining images and quantification of intracellular lipid accumulation in PC-3 and DU145 cells under the indicated genetic and pharmacological conditions. For quantified data, results are presented as mean ± SD from three independent biological replicates (n = 3). Statistical significance was assessed using one-way ANOVA within each genetic background (Lv-Ctrl or Lv-OE-CCT2) for each cell line. ns indicates P > 0.05, *P < 0.05, **P < 0.01, ***P < 0.001, ****P < 0.0001.

**Supplementary Figure 10. The CCT2-EIF3F-FASN axis is associated with metastatic phenotypes and bone metastatic burden in prostate cancer models.**

(A) Co-immunoprecipitation (Co-IP) assays showing endogenous interactions among CCT2, EIF3F, and FASN in RM-1 cells. (B) Immunoblot analysis of FASN, CCT2, and EIF3F protein levels in RM-1 cells transduced with plvx-vector or plvx-CCT2, with or without EIF3F knockdown (si-EIF3F). (C) Quantification of EIF3F, CCT2, and FASN protein level, normalized to β-actin and expressed relative to the control group. Data are presented as mean ± SD from three independent biological replicates (n = 3). Statistical significance was assessed using one-way ANOVA. (D) Ubiquitination assay of FASN in RM-1 cells. Cells were transfected with His-ubiquitin and subjected to Ni-NTA pull-down followed by immunoblotting for FASN. Whole-cell lysates (WCL) show the expression of FASN, CCT2, EIF3F, and β-actin. (E) Immunoblot analysis of EMT-related markers (Vimentin, E-cadherin, N-cadherin, SLUG, and SNAI1) in RM-1 cells expressing plvx-vector or plvx-CCT2, with or without EIF3F knockdown and treatment with Orlistat or Y043-8015, as indicated. (F) Quantification of EMT marker expression. Data are presented as mean ± SD from three independent biological replicates (n = 3). Statistical significance was assessed using one-way ANOVA. (G) Wound-healing assays (upper panels) and Transwell migration and invasion assays (lower panels) in RM-1 cells expressing plvx-vector or plvx-CCT2, with additional treatment of si-EIF3F, Orlistat, or Y043-8015 as indicated. (H) Quantification analysis of cell migration distance, migrated cell number, and invaded cell number. Data are presented as mean ± SD from three independent biological replicates (n = 3). Statistical significance was assessed using one-way ANOVA. (I) Representative images of excised tumors from mice treated with Y043-8015 at the indicated doses, showing dose-dependent reduction in tumor growth. (J) Body weight curves of mice treated with vehicle or Y043-8015 (40 mg/kg, IP) under the same regimen used in the efficacy studies. Data are presented as mean ± SD, *n* = 5 mice per group. Statistical significance was assessed using two-way ANOVA. (K) Representative H&E staining images of major organs (heart, liver, spleen, lung, and kidney) collected from vehicle- and Y043-8015-treated mice at the endpoint. (L) Representative micro-CT images of hind limbs from mice bearing RM-1 tumors with control or CCT2 overexpression (Lv-Ctrl or Lv-OE-CCT2), treated with vehicle, Orlistat, Y043-8015, or the combination, showing representative bone architecture and osteolytic lesions. (M) Representative immunofluorescence staining of bone metastatic lesions for EMT-related markers under the indicated genetic and pharmacological conditions. Quantification of fluorescence intensity is shown on the right. Data are presented as mean ± SD from three independent biological replicates (n = 3). Statistical analysis was performed using one-way ANOVA within each background (Lv-Ctrl or Lv-OE-CCT2). (N) Violin plot showing normalized CCT2 expression levels (FPKM) in primary prostate cancer (PCa) tissues and bone metastasis samples from the SU2C/PCF cohort. Statistical significance was assessed using two-tailed unpaired Student’s *t*-tests. For quantitative panels with significance annotations, ns indicates P > 0.05, *P < 0.05, **P < 0.01, ***P < 0.001, ****P < 0.0001.

**Supplementary Tables**
